# Supplementary material for: β-Lactam TRPM8 Antagonists Derived from Phe-Phenylalaninol Conjugates: Structure–Activity Relationships and Antiallodynic Activity
Source: Int J Mol Sci. 2023 Oct 4;24(19):14894. doi: 10.3390/ijms241914894 (PMC10573892; doi:10.3390/ijms241914894)
Supplement: Supplementary file 1 [file ijms-24-14894-s001.zip › ijms-2614934-supplementary.pdf]

β-Lactam TRPM8 antagonists derived from Phe-phenylalaninol conjugates: structure-activity relationships and antiallodynic activity

Cristina Martín-Escura, Mª Angeles Bonache, Jessy Medina, Alicia Medina-Peris, Jorge De Andrés-López, Sara González-Rodríguez, Sara Kerselaers, Gregorio Fernández-Ballester, Thomas Voets, Antonio Ferrer-Montiel, Asia Fernández-Carvajal, Rosario González-Muñiz

Supplementary Materials

1- Calculated TPSA Values S2
2- Synthetic Methods, Intermediates, and Diastereoisomeric β-Lactam Characterization S6
3- Molecular Modeling: protocols and detailed interactions S26

Table S1. Total Polar Surface Area (TPSA) calculated for new β–lactam derivatives and model **1**.

Compound	Formula	[a]TPSA, A^2
1	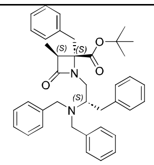	49.85
4	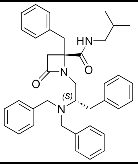	52.65
5	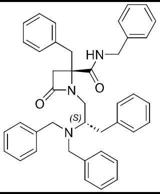	52.65
6	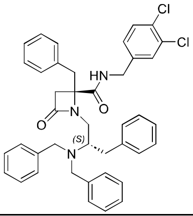	52.65
7	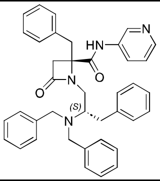	65.01
8	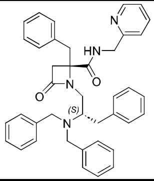	65.01
9	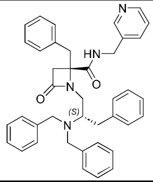	65.01
13	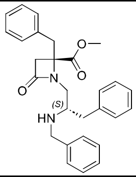	58.64
14	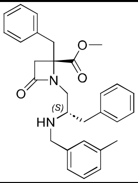	58.64

15	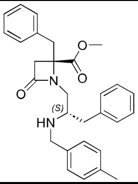	58.64
16	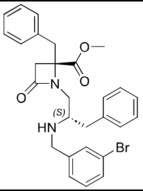	58.64
17	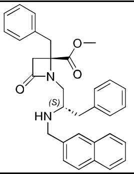	58.64
18	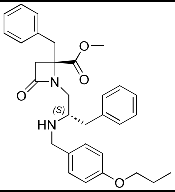	67.87
19	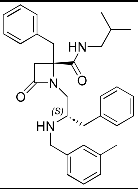	61.44
20	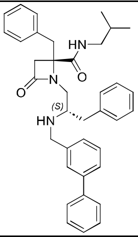	61.44
21	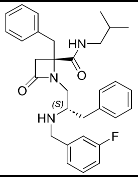	61.44
22	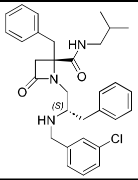	61.44
23	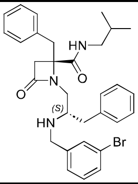	61.44

24	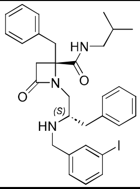	61.44
25	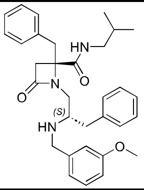	70.67
26	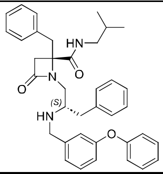	70.67
27	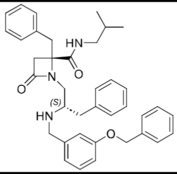	70.67
28	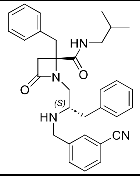	85.23
29	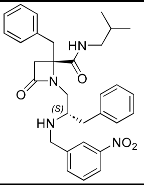	113.25
30	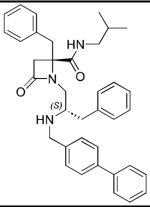	61.44
31	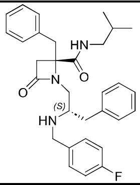	61.44
32	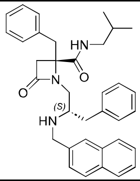	61.44

34	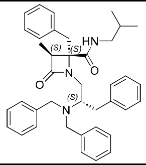	52.65
35	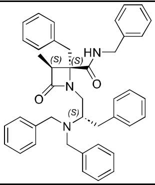	52.65
36	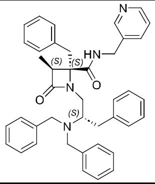	65.01
37	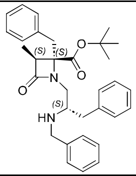	58.64
40	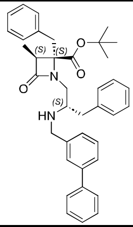	58.64
41	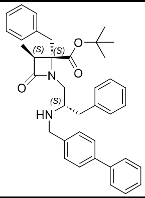	58.64
42	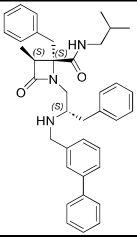	61.44
43	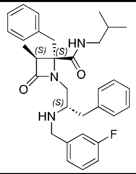	61.44
44	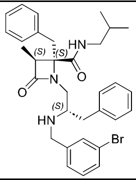	61.44

45	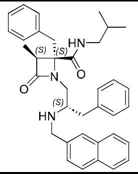	61.44

[a] Calculated with ChemDraw 22.2.0.

Synthetic Methods

General procedures: Reactions were monitored either by TLC and/or analytic HPLC (UV, detection, 220 nm). Flash columns, filled with silica gel Merck 60 (230–400) were used for chromatographic separations. A reversed-phase column, Sunfire C18 (Waters, Barcelona, Spain; 4.6 × 50 mm, 3.5 µm), a flux of 1 mL/min, and mixtures of CH_3CN, phase A, and H_2O, phase B, both containing 0.01% formic acid, were used for analytical HPLCs. Mass spectra, in electrospray, positive mode, were obtained on a Waters Micromass ZQ spectrometer (Waters, Barcelona, Sapin). High resolution mass spectrum (ESI-HRMS) was recorded on an Agilent 6520 Q-TOF instrument (Agilent, Madrid, Spain). Optical rotation for final compounds was measured in a polarimeter Perkin Elmer 141 apparatus (Perkin Elmer España, SL, Madrid, Spain). NMR spectra were recorded in a Varian INOVA-400 (400 MHz) spectrometer or Brucker 300 (Brucker Española SA, Rivas-Vaciamadrid, Spain), operating at 400 and 75 MHz for ^1H and ^{13}C experiments, respectively (with chemical shifts expressed in ppm and coupling constants in Hz). Bidimensional COSY and HSQC experiments were used for assignments when required. Reactives: HCl/Dioxano, PyBroP, TEA, BTPP, aldehydes, amines and dry solvents were acquired from Merck KGaA (Madrid, Spain). Solvents: HPLC grade MeOH, EtOAc, dichloromethane, acetonitrile were purchased from Symta SLL (Madrid, Spain). β–Lactam derivatives derivatives **1** and **2a,b** were prepared as described [1,2].

General procedures for the synthesis of carboxylic acid intermediates

Hydrolysis of methyl esters: To a solution of the corresponding substitute β-lactam 4-alkoxycarbonyl (1.71 mmol) in MeOH (20 mL) is added 2M NaOH (2.57 mmol, 1.21 mL). Then, the reaction mixture is stirred at room temperature. Once disappearance of the starting product, the solvent is evaporated to dryness. The resulting residue is dissolved in EtOAc:H_2O (1:1), separating the phases and the aqueous phase is brought to pH 3 with 1M HCl and extracted with EtOAc. The organic phase is washed with saturated NaCl solution, dried over anhydrous Na_2SO_4, filtered and evaporated to dryness. The resulting crude is purified on a silica gel column, using the eluent system indicated in each case.

Hydrolysis of tert-butyl esters: To a solution of the corresponding substituted β-lactam 4-*terc*-butoxycarbonyl (1.019 mmol) in DCM (5 mL) is added 4M HCl/Dioxano (10.19 mmol, 2.5 mL) and the reaction mixture is stirred at room temperature. Once completed, the solvent is evaporated to dryness. The resulting crude is purified on a silica gel column, using the eluent system indicated in each case.

General procedure for the synthesis of substituted 4-carboxamides . To a solution of β-lactam 4-carboxylate (0.188 mmol) in dry DCM (5 mL) is added PyBrOP (0.225 mmol, 0.105 g), TEA (0.225 mmol, 0.031 mL) and the corresponding amine (0.225 mmol). The reaction is stirred at room temperature, and after disappearance of the starting product, the solvent is evaporated to dryness. The crude obtained is dissolved in EtOAc, washed with 0.1 M HCl, $NaHCO_3$ (10%) and

saturated NaCl solution. The organic phase is dried over anhydrous Na_2SO_4, filtered and evaporated to dryness. The resulting crude is purified on a silica gel column, using the eluent system indicated in each case.

General procedure for N-debenzylation. A solution of the corresponding β-lactam derivative with NBn_2 (1.17 mmol) in MeOH (20 mL) is cooled to 0 °C and 10 wt.% $Pd(OH)_2$ is slowly added followed by 1M HCl (1.17 mL, 1.17 mmol). The mixture is hydrogenated in a Parr at 45-47 psi H_2 and 40 ºC for 4 h. The catalyst is removed by filtration and the solvent is evaporated to dryness. The resulting crude is purified on a silica gel column, using the eluent system indicated in each case.

General procedure for Synthesis of 2'-N-monobenzyl derivatives. To a solution of the corresponding substituted β-lactam 2'-NH_2 derivative (0.225 mmol) in MeOH (4 mL) is added TEA (0.225 mmol, 0.031 mL) and the corresponding aldehyde (0.337 mmol). The reaction mixture is stirred for 1.5 h at room temperature. Once the imine is formed, $NaBH_4$ (0.450 mmol, 0.017 g) is added at 0 ºC and stirred at room temperature. After 24 hours of reaction, the solvent is evaporated to dryness. The organic residue is dissolved in EtOAc and washed with H_2O and saturated NaCl solution successively. The organic phase is dried over anhydrous Na_2SO_4, filtered and evaporated to dryness. The resulting crude is purified on a silica gel column, using the eluent system indicated in each case.

4*R*,*S*-Benzyl-4-carboxy-1-[(2'*S*-dibenzylamino-3'-phenyl)prop-1'-yl]-2-oxoazetidine (3a,b)

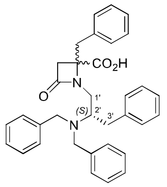

White solid. Yield: 90% (From **2a,b**). Eluent: 2% to 9% of MeOH in DCM. HPLC: t_R = 7.02 (m, 3a) y 7.22 (M, 3b) min (gradient from 20% to 95% of A in 10 min). Ratio of diastereoisomers M:m, 1.8:1.^{1}H-NMR (300 MHz, $CDCl_3$, major diastereoisomer **3b**, from the mixture): *δ* 7.61 - 7.35 (m, 12H, Ar), 7.33 - 7.04 (m, 4H, Ar), 6.98 (d, *J* = 7.5 Hz, 1H, Ar), 6.86 (t, *J* = 7.4 Hz, 1H, Ar), 6.70 (t, *J* = 7.6 Hz, 1H, Ar), 6.42 (d, *J* = 7.3 Hz, 1H, Ar), 4.38 (s, 2H, NCH_2), 3.93 (d, *J* = 12.2 Hz, 1H, 4-CH_2), 3.84 (dd, *J* = 14.9, 10.3 Hz, 1H, $H_{1'}$), 3.63 (d, *J* = 13.1 Hz, 2H, NCH_2), 3.33 (d, *J* = 15.1 Hz, 1H, H_3), 3.12 (dd, *J* = 13.0, 3.4 Hz, 1H, $H_{3'}$), 3.06 (d, *J* = 15.1 Hz, 1H, H_3), 2.82 - 2.64 (m, 3H, 4-CH_2, $H_{2'}$, $H_{1'}$), 2.55 (m, 1H, $H_{3'}$). ^{13}C-NMR (75 MHz, $CDCl_3$): *δ* 175.8 (COOH), 169.7 (C_2), 136.5, 130.9, 130.2, 129.4, 129.2, 129.2, 129.0, 128.9, 128.8, 128.7, 127.2, 126.9 (Ar), 66.5 (C_4), 55.4 ($C_{2'}$), 54.1 (NCH_2), 50.8 (C_3), 41.3 ($C_{1'}$), 40.6 (4-CH_2), 32.4 ($C_{3'}$). ^{1}H-NMR (300 MHz, $CDCl_3$, minor diastereoisomer **3a**, from the mixture): *δ* 7.61 - 7.35 (m, 13H, Ar), 7.33 - 7.04 (m, 4H, Ar), 6.98 (d, *J* = 7.5 Hz, 1H, Ar), 6.70 (t, *J* = 7.6 Hz, 1H, Ar), 6.42 (d, *J* = 7.3 Hz, 1H, Ar), 3.93 (m, 3H, NCH_2, $H_{2'}$), 3.61 (d, *J* = 13.1 Hz, 2H, NCH_2), 3.21 (dd, *J* = 16.0, 2.6 Hz, 1H, $H_{1'}$), 3.08 (m, 1H, $H_{3'}$), 3.01(d, *J* = 14.9 Hz, 1H, H_3), 2.88 (d, *J* = 13.8 Hz, 1H, 4-CH_2), 2.82 - 2.64 (m, 3H, H_3, $H_{1'}$, 4-CH_2), 2.60 - 2.45 (m, 1H, $H_{3'}$). ^{13}C-NMR (75 MHz, $CDCl_3$): *δ* 175.6 (COOH), 167.2 (C_2), 136.9, 134.4, 131.1, 130.7, 129.8, 129.6, 129.5, 129.0, 128.9, 128.7, 128.1, 127.6 (Ar), 65.5 (C_4), 59.7 ($C_{2'}$), 54.1 (NCH_2), 48.6 (C_3), 41.7 ($C_{1'}$), 40.8 (4-CH_2), 29.8 ($C_{3'}$). MS(ES)$^+$: 519.31 $[M+H]^+$.

4*R*,*S*-Benzyl-4-[*N*-(*iso*-butyl)carbamoyl]-1-[(2'*S*-dibenzylamino-3'-phenyl)prop-1'-yl]-2-oxo-azetidine (4a,b)

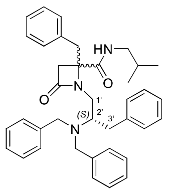

Syrup. Yield: 92% (From **3a,b** and *iso*-butylamine). Eluent: 16% to 33% of EtOAc in hexane . HPLC: t_R = 7.57 (m, a) y 8.43 (M, b) min (gradient from 15% to 95% of A in 10 min). Ratio of diastereoisomers M:m, 1.6:1. ^{1}H-NMR (300 MHz, $CDCl_3$, major diastereoisomer **4b**, from the mixture): δ 7.40 - 7.00 (m, 20H, Ar), 5.51 (t, J = 5.9 Hz, 1H, NH), 3.90 (dd, J = 14.2, 4.7 Hz, 1H, $H_{1'}$), 3.80 (d, J = 13.8 Hz, 2H, NCH_2), 3.70 (d, J = 13.8 Hz, 2H, NCH_2), 3.50 (dd, J = 14.2, 9.1 Hz, 1H, $H_{1'}$), 3.27 (m, 1H, $H_{2'}$), 3.10 (d, J = 13.6 Hz, 1H, 4-CH_2), 2.98 - 2.85 (m, 5H, H_3, $H_{3'}$ y CH_2, ^{i}Bu), 2.83 (m, 1H, $H_{3'}$), 2.47 (d, J = 13.6 Hz, 1H, 4-CH_2), 1.54 (m, 1H, CH, ^{i}Bu), 0.76 (d, J = 6.7 Hz, 3H, CH_3, ^{i}Bu), 0.72 (d, J = 6.7 Hz, 3H, CH_3, ^{i}Bu). ^{13}C-NMR (75 MHz, $CDCl_3$): δ 170.4 (4-CONH), 166.6 (C_2), 140.0, 139.5, 135.4, 129.6, 129.4, 129.0, 128.8, 128.3, 127.5, 127.1, 126.2 (Ar), 64.2 (C_4), 59.0 ($C_{2'}$), 53.3 (NCH_2), 47.2 (C_3), 46.2 (CH_2, ^{i}Bu), 42.0 ($C_{1'}$), 40.8 (4-CH_2), 36.6 ($C_{3'}$), 28.3 (CH, ^{i}Bu), 20.2 (CH_3, ^{i}Bu). ^{1}H-NMR (300 MHz, $CDCl_3$, minor diastereoisomer **4a**, from the mixture): δ 7.40 - 6.90 (m, 20H, Ar), 5.30 (t, J = 5.9 Hz, 1H, NH), 3.77 (d, J = 13.5 Hz, 2H, NCH_2), 3.60 (d, J = 13.7 Hz, 2H, NCH_2), 3.46 (dd, J = 14.1, 9.0 Hz, 1H, $H_{1'}$), 3.24 (m, 2H, $H_{2'}$, $H_{1'}$), 3.00 (dd, J = 14.8, 5.9 Hz, 1H, $H_{3'}$), 2.98 - 2.85 (m, 4H, H_3, CH_2, ^{i}Bu), 2.74 (d, J = 13.9 Hz, 1H, 4-CH_2), 2.62 (m, 1H, $H_{3'}$), 2.41 (d, J = 14.1 Hz, 1H, 4-CH_2), 1.41 (m, 1H, CH, ^{i}Bu), 0.65 (d, J = 6.7 Hz, 3H, CH_3, ^{i}Bu), 0.63 (d, J = 6.7 Hz, 3H, CH_3, ^{i}Bu). ^{13}C-NMR (75 MHz, $CDCl_3$): δ 170.3 (4-CONH), 166.6 (C_2), 140.0, 139.6, 135.6, 129.5, 129.3, 128.9, 128.6, 128.4, 127.3, 127.2, 126.4 (Ar), 63.7 (C_4), 60.3 ($C_{2'}$), 53.6 (NCH_2), 47.0 (C_3), 46.9 (CH_2, ^{i}Bu), 42.02 ($C_{1'}$), 39.6 (4-CH_2), 34.7 ($C_{3'}$), 28.3 (CH, ^{i}Bu), 20.1, 20.0 (CH_3, ^{i}Bu). MS(ES)$^+$: 574.42 $[M+H]^+$. Exact mass calculated for $C_{38}H_{43}N_3O_2$: 573.33553, found 573.33717.

4*R*,*S*-Benzyl-4-[*N*-(benzyl)carbamoyl]-1-[(2'*S*-dibenzylamino-3'-phenyl)prop-1'-yl]-2-oxo-azetidine (5a,b)

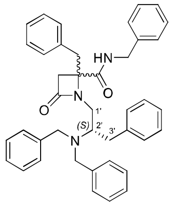

Syrup. Yield: 38% (From **3a,b** and benzylamine). Eluent: 10% to 30% of EtOAc in DCM. HPLC: t_R = 7.72 (m, 2.11a) y 8.87 (M, 2.11b) min (gradient from 15% to 95% of A in 10 min). Ratio of diastereoisomers M:m, 2.6:1.^{1}H-NMR (300 MHz, $CDCl_3$, major diastereoisomer **5b**, from the mixture): δ 7.30 - 6.95 (m, 25H, Ar), 6.10 (t, J = 5.7 Hz, 1H, NH), 4.24 (d, J = 5.7 Hz, 2H, NHCH_2, Bn), 3.84 (dd, J = 14.3, 5.2 Hz, 1H, $H_{1'}$), 3.74 (d, J = 13.5 Hz, 2H, NCH_2) 3.67 (d, J = 13.8 Hz, 2H, NCH_2), 3.42 (dd, J = 14.2, 8.8 Hz, 1H, $H_{1'}$), 3.24 (m, 1H, $H_{2'}$), 3.12 (d, J = 13.7 Hz, 1H, 4-CH_2), 2.95 (d, J = 14.9 Hz, 1H, H_3), 2.91 (d, J = 14.7 Hz, 1H, H_3), 2.86 (dd, J = 13.4, 9 Hz, 1H, $H_{3'}$), 2.72 (dd, J = 13.8, 6.2 Hz, 1H, $H_{3'}$), 2.61 (d, J = 13.7 Hz, 1H, 4-CH_2). ^{13}C-NMR (75 MHz, $CDCl_3$): δ 170.5 (4-CONH), 166.8 (C_2), 139.9, 139.3, 137.5, 135.2, 129.7, 129.4, 129.0, 128.9, 128.8, 128.4, 128.0, 127.8, 127.4, 127.1, 126.2 (Ar), 64.2 (C_4), 59.1 ($C_{2'}$), 53.2 (NCH_2), 46.3 (C_3), 43.9 ($NHCH_2$, Bn), 42.0 ($C_{1'}$), 40.6 (4-CH_2), 36.4 ($C_{3'}$). ^{1}H-NMR (300 MHz, $CDCl_3$, minor diastereoisomer **5a**, from the mixture): δ 7.30 - 6.90 (m, 25H, Ar), 6.15 (t, J = 5.7 Hz, 1H, NH), 4.14 (dd, J = 14.7, 6.0 Hz, 1H, NHCH_2, Bn), 3.73 (m, 1H, NHCH_2, Bn), 3.72 (d, J = 13.5 Hz, 2H, NCH_2), 3.55 (d, J = 13.5 Hz, 2H, NCH_2), 3.34 (m, 2H, $H_{2'}$, $H_{1'}$), 3.00 (dd, J = 13.8, 5.3 Hz, 1H, $H_{3'}$), 2.96 (m, 1H, H_3), 2.93 (m, 1H, $H_{1'}$), 2.85 (d, J = 14.0

Hz, 1H, 4-CH_2), 2.84 (d, J = 12.8 Hz, 1H, H_3), 2.60 (d, J = 14.0 Hz, 1H, 4-CH_2) 2.53 (dd, J = 13.8, 8.5 Hz, 1H, $H_{3'}$). ^{13}C-NMR (75 MHz, $CDCl_3$): δ 170.5 (4-CONH), 166.6 (C_2), 139.7, 139.2, 137.7, 135.6, 129.6, 129.4, 128.8, 128.7, 128.6, 128.4, 128.0, 127.8, 127.6, 127.3, 126.4 (Ar), 63.8 (C_4), 60.1 ($C_{2'}$), 53.8 (NCH_2), 47.1 (C_3), 43.4 ($NHCH_2$, Bn), 42.5 ($C_{1'}$), 39.8 (4-CH_2), 34.3 ($C_{3'}$). MS(ES)$^+$: 608.26 $[M+H]^+$. Exact mass calculated for $C_{41}H_{41}N_3O_2$: 607.31988, found 607.31848.

4*R*-Benzyl-4-[*N*-(3,4-dichlorobenzyl)carbamoyl]-1-[(2'*S*-dibenzylamino-3'- phenyl)prop -1'-yl]-2-oxoazetidine (6a)

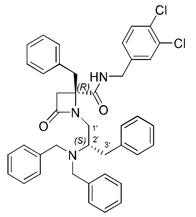

Syrup. Yield: 15% (From **3a,b** and 3,4-dichlorobenzylamine). Eluent: 3% to EtOAc in DCM. HPLC: t_R = 8.58 min (gradient from 30% to 95% of A in 10 min). ^{1}H-NMR (300 MHz, $CDCl_3$): δ 7.53 - 6.69 (m, 23H, Ar), 6.46 (t, J = 5.9 Hz, 1H, NH), 4.14 (dd, J = 14.9, 6.0 Hz, 1H, NHCH_2, Bn), 4.07 (dd, J = 14.9, 5.8 Hz, 1H, NHCH_2, Bn), 3.86 (dd, J = 14.5, 6.3 Hz, 1H, $H_{1'}$), 3.74 (s, 4H, 2 NCH_2), 3.34 (dd, J = 14.5, 7.7 Hz, 1H, $H_{1'}$), 3.20 (m, 1H, $H_{2'}$), 3.11 (d, J = 13.9 Hz, 1H, 4-CH_2), 2.96 (s, 2H, H_3), 2.87 (dd, J = 13.9, 6.7 Hz, 1H, $H_{3'}$), 2.72 (d, J = 13.9 Hz, 1H, 4-CH_2), 2.63 (dd, J = 13.9, 7.2 Hz, 1H, $H_{3'}$). ^{13}C-NMR (75 MHz, $CDCl_3$): δ 171.1 (4-CONH), 167.0 (C_2), 139.7, 139.1, 138.0, 135.1, 132.8, 131.8, 130.7, 129.9, 129.7, 129.3, 129.1, 128.9, 128.5, 128.5, 127.6, 127.3, 127.3, 126.3 (Ar), 64.2 (C_4), 59.6 ($C_{2'}$), 53.2 (NCH_2), 46.9 (C_3), 42.7 (NCH_2, Bn), 42.0 ($C_{1'}$), 40.0 (4-CH_2), 36.1 ($C_{3'}$). MS(ES)$^+$: 676.63 $[M+H]^+$, 678.62 $[M+2]^+$, 680.61 $[M+4]^+$. Exact mass calculated for $C_{41}H_{39}Cl_2N_3O_2$: 675.24193, found 675.24237.

4*R*,*S*-Benzyl-4-[*N*-(3,4-dichlorobenzyl)carbamoyl]-1-[(2'*S*-dibenzylamino-3'- phenyl)prop -1'-yl]-2-oxoazetidine (6a,b)

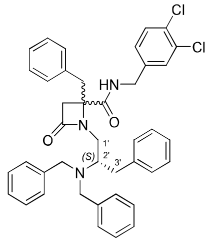

Syrup. Yield: 46% (From **3a,b** and 3,4-dichlorobenzylamine). Eluent: 3% to EtOAc in DCM. HPLC: t_R = 6.82 (M, **6b**) y 8.33 (m, **6a**) min (gradient from 30% to 95% of A in 10 min). Ratio of diastereoisomers M:m, 1.4:1. ^{1}H-NMR (300 MHz, $CDCl_3$, major diastereoisomer **6b**, from the mixture): δ 7.39 - 6.93 (m, 23H, Ar), 6.75 (s, 1H, NH), 4.10 (m, 2H, NHCH_2, Bn), 3.86 (dd, J = 14.3, 6.2 Hz, 1H, $H_{1'}$), 3.77 (s, 4H, NCH_2), 3.25 (dd, J = 14.5, 3.7 Hz, 1H, $H_{1'}$), 3.16 (m, 3H, $H_{2'}$, 4-CH_2, $H_{3'}$), 3.12 (d, J = 14.5 Hz, 1H, H_3), 2.84 (d, J = 14.7 Hz,1H, H_3), 2.77 (m, 1H, 4-CH_2), 2.75 (dd, J = 13.9, 7.3 Hz, 1H, $H_{3'}$). ^{13}C-NMR (75 MHz, $CDCl_3$): δ 171.1 (4-CONH), 166.6 (C_2), 138.2, 135.0, 132.6, 131.6, 130.6, 129.9, 129.7, 129.6, 129.4, 129.3, 129.2, 128.9, 128.8, 128.6, 128.5, 127.6, 127.3, 126.4 (Ar), 63.9 (C_4), 59.6 ($C_{2'}$), 54.1 (NCH_2), 47.5 (C_3), 42.8 ($NHCH_2$, Bn), 42.0 ($C_{1'}$), 40.1 (4-CH_2), 33.8 ($C_{3'}$). MS(ES)$^+$: 676.63 $[M+H]^+$, 678.62 $[M+2]^+$, 680.61 $[M+4]^+$.

4*R*,*S*-Benzyl-4-[*N*-(3''-pyridyl)carbamoyl]-1-[(2'*S*-dibenzylamino-3'-phenyl)prop-1'-yl]-2-oxoazetidine (7a,b)

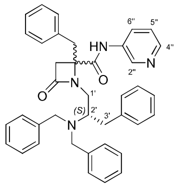

Syrup. Yield: 85% (From **3a,b** and 3-aminopyridine). Eluent: 1% to 10% of MeOH in DCM. HPLC: t_R = 6.65 (m, **7a**) y 7.07 (M, **7b**) min (gradient from 15% to 95% of A in 10 min). Ratio of diastereoisomers M:m, 1.4:1. ^{1}H-NMR (300 MHz, $CDCl_3$, major diastereoisomer **7b**, from the mixture): δ 8.30 (m, 1H, Ar), 8.02 (s, 1H, NH), 7.65 - 6.90 (m, 23H, Ar), 3.97 (dd, *J* = 14.5, 6.3 Hz, 1H, $H_{1'}$), 3.75 (s, 4H, NCH_2), 3.37 (m, 2H, $H_{1'}$), 3.24 (m 1H, $H_{2'}$), 3.14 (d, *J* = 13.9 Hz, 1H, 4-CH_2), 3.08 (s, 2H, H_3), 2.92 (dd, *J* = 13.9, 6.8 Hz, 1H, $H_{3'}$), 2.67 (dd, *J* = 14.1, 7.2 Hz, 1H, $H_{3'}$), 2.56 (d, *J* = 13.7 Hz, 1H, 4-CH_2). ^{13}C-NMR (75 MHz, $CDCl_3$): δ 170.1 (4-CONH), 167.1 (C_2), 146.2, 142.4, 139.5, 138.9, 135.0, 133.7, 129.7, 129.4, 129.4, 129.2, 128.5, 128.5, 127.7, 127.4, 126.3, 123.6 (Ar), 64.7 (C_4), 58.9 ($C_{2'}$), 53.5 (NCH_2), 47.3 (C_3), 42.3 ($C_{1'}$), 40.4 (4-CH_2), 35.8 ($C_{3'}$). ^{1}H-NMR (300 MHz, $CDCl_3$, minor diastereoisomer **7a**, from the mixture): δ 7.89 (s, 1H, NH), 7.39 - 7.00 (m, 24H, Ar), 3.80 (d, *J* = 13.6 Hz, 2H, NCH_2), 3.60 (d, *J* = 13.4 Hz, 2H, NCH_2), 3.54 (m, 1H, $H_{1'}$), 3.37 (m, 1H, $H_{1'}$), 3.32 (m 1H, $H_{2'}$), 3.04 (d, *J* = 14.5 Hz, H_3), 2.95 (d, *J* = 14.5 Hz, H_3), 2.92 - 2.80 (m, 3H, $H_{3'}$, 4-CH_2), 2.60 (m, 1H, $H_{3'}$). ^{13}C-NMR (75 MHz, $CDCl_3$): δ 169.8 (4-CONH), 166.4 (C_2), 146.0, 142.2, 139.7, 139.2, 135.4, 133.5, 129.7, 129.3, 129.1, 128.6, 128.4, 128.3, 127.8, 127.3, 126.4, 123.5 (Ar), 64.2 (C_4), 60.2 ($C_{2'}$), 53.9 (NCH_2), 47.2 (C_3), 42.4 ($C_{1'}$), 40.0 (4-CH_2), 34.3 ($C_{3'}$). MS(ES)$^+$: 595.40 $[M+H]^+$. Exact mass calculated for $C_{39}H_{38}N_4O_2$: 594.29948, found 594.30209.

4*R*,*S*-Benzyl-4-[*N*-(2''-pyridyl)methyl)carbamoyl]-1-[(2'*S*-dibenzylamino-3'- phenyl) prop-1'-yl]-2-oxoazetidine (8a,b)

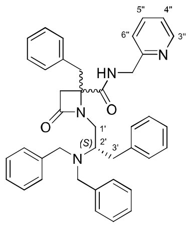

Syrup. Yield: 51% (From**3a,b** and 2-picolylamine). Eluent: 0.9% of MeOH in DCM. HPLC: t_R = 6.75 (m, **8a**) y 7.31(M,**8b**) min (gradient from 15% to 95% of A in 10 min). Ratio of diastereoisomers M:m, 1.8:1. ^{1}H-NMR (300 MHz, $CDCl_3$, major diastereoisomer **2.14b**, from the mixture): δ 8.42 (s, 1H, NH), 7.64 (td, *J* = 7.7, 1.8 Hz, 1H, $H_{4''}$), 7.38 - 6.89 (m, 23H, Ar), 4.41 (s, 2H, NHC*H*$_2$), 3.86 (dd, *J* = 13.8, 4.3 Hz, 1H, $H_{1'}$), 3.78 (s, 4H, NCH_2), 3.50 - 3.30 (m, 4H, $H_{1'}$, $H_{2'}$), 3.19 (d, *J* = 13.8 Hz, 1H, 4-CH_2), 3.12 (d, *J* = 14.9 Hz, 1H, H_3), 2.98 (d, *J* = 14.8 Hz, 1H, H_3), 2.84 (dd, *J* = 14.0, 5.8 Hz, 1H, $H_{3'}$), 2.66 (d, *J* = 13.6 Hz, 1H, 4-CH_2). ^{13}C-NMR (75 MHz, $CDCl_3$): δ 170.5 (4-CONH), 166.9 (C_2), 155.8, 149.3, 139.7, 137.2, 135.5, 130.0, 129.9, 129.8, 129.3, 129.0, 128.6, 127.6, 127.4, 126.4, 122.9, 122.3 (Ar), 64.2 (C_4), 58.7 ($C_{2'}$), 53.3 (NCH_2), 48.6 (C_3), 44.3 ($C_{1'}$), 42.0 ($NHCH_2$), 39.5 (4-CH_2), 36.5 ($C_{3'}$). ^{1}H-NMR (300 MHz, $CDCl_3$, minor diastereoisomer **2.14a**, from the mixture): δ 8.43 (s, 1H, NH), 7.38 - 6.89 (m, 24H, Ar), 4.42 (s, 2H, NHC*H*$_2$), 3.80 (d, *J* = 13.5 Hz, 2H, NCH_2), 3.75 (m, 1H, $H_{1'}$), 3.68 (m, 3H, NCH_2, $H_{2'}$), 3.50 - 3.30 (m, 2H, $H_{1'}$, NHC*H*$_2$), 2.97 (m, 3H, $H_{3'}$, 4-CH_2), 2.66 (m, 1H, $H_{3'}$). ^{13}C-NMR (75 MHz, $CDCl_3$): δ 170.5 (4-CONH), 166.7 (C_2), 155.7, 155.5, 149.1, 139.5, 136.9, 135.2, 129.6, 129.5, 128.8, 128.7, 128.3, 127.3, 127.1, 126.2, 122.6, 121.9 (Ar), 63.7

(C_4), 58.8 ($C_{2'}$), 53.6 (NCH_2), 48.5 (C_3), 46.0 ($C_{1'}$), 40.8 ($NHCH_2$), 39.4 (4-CH_2), 36.4 ($C_{3'}$). MS(ES)$^+$: 609.56 $[M+H]^+$. Exact mass calculated for $C_{40}H_{40}N_4O_2$: 608.31513, found 608.31523.

4*R*,*S*-Benzyl-4-[*N*-(3''-pyridyl)methyl)carbamoyl]-1-[(2'*S*-dibenzylamino-3'- phenyl)prop-1'-yl]-2-oxoazetidine (9a,b)

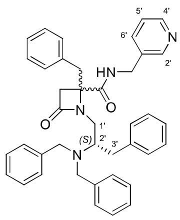

Syrup. Yield: 37% (From **3a,b** and 3-picolylamine). Eluent: 1% to 10% of EtOAc in DCM. HPLC: t_R = 6.08 (m, **9a**) y 6.54 (M, **9b**) min (gradient from 15% to 95% of A in 10 min). Ratio of diastereoisomers M:m, 1.2:1. ^{1}H-NMR (300 MHz, $CDCl_3$, major diastereoisomer **9b**, from the mixture): δ 8.51 (dd, J = 4.8, 1.7 Hz, 1H, $H_{4''}$), 8.29 (s, 1H, $H_{2''}$), 7.40 - 6.87 (m, 22H, Ar), 6.46 (t, J = 6.2 Hz, 1H, NH), 4.22 (dd, J = 14.9, 6.0 Hz, 1H, $NHCH_2$), 4.11 (dd, J = 14.8, 5.5 Hz, 1H, $NHCH_2$), 3.87 (dd, J = 14.4, 5.8 Hz, 1H, $H_{1'}$), 3.74 (s, 4H, NCH_2), 3.36 (m, 2H, $H_{1'}$), 3.23 (m, 1H, $H_{2'}$), 3.12 (d, J = 13.9 Hz, 1H, 4-CH_2), 2.98 (s, 2H, H_3), 2.89 (m, 1H, $H_{3'}$), 2.68 (dd, J = 13.5, 5.1 Hz, 1H, $H_{3'}$), 2.65 (d, J = 14.3 Hz, 4-CH_2). ^{13}C-NMR (75 MHz, $CDCl_3$): δ 170.0 (4-CONH), 166.7 (C_2), 147.9, 147.7, 138.5, 138.1, 134.8, 133.9, 132.3, 128.5, 128.0, 127.8, 127.6, 127.3, 126.4, 125.2, 122.6 (Ar), 63.9 (C_4), 59.5 ($C_{2'}$), 53.3 (NCH_2), 47.5 (C_3), 42.0 ($C_{1'}$), 41.3 ($NHCH_2$), 40.2 (4-CH_2), 36.1 ($C_{3'}$). ^{1}H-NMR (300 MHz, $CDCl_3$, minor diastereoisomer **9a**, from the mixture): δ 8.44 (dd, J = 4.8, 1.7 Hz, 1H, $H_{4''}$), 8.17 (s, 1H, $H_{2''}$), 7.40 - 6.87 (m, 22H, Ar), 6.46 (t, J = 5.6 Hz, 1H, NH), 3.76 (d, J = 13.3 Hz, 2H, NCH_2), 3.56 (d, J = 13.3 Hz, 2H, NCH_2), 3.36 (m, 2H, $H_{1'}$), 3.38 (m, 2H, $H_{2'}$, $NHCH_2$), 3.23 (m, 3H, $NHCH_2$, $H_{1'}$), 3.00 (m, 2H, $H_{3'}$, 4-CH_2), 2.85 (s, 2H, H_3), 2.66 (m, 1H, 4-CH_2), 2.50 (dd, J = 13.6, 8.7 Hz, 1H, $H_{3'}$). ^{13}C-NMR (75 MHz, $CDCl_3$): δ 171.1 (4-CONH), 167.0 (C_2), 147.7, 147.5, 138.6, 137.8, 134.6, 134.4, 132.5, 128.3, 128.2, 127.7, 127.6, 127.3, 126.1, 125.4, 122.5 (Ar), 64.2 (C_4), 60.2 ($C_{2'}$), 54.0 (NCH_2), 46.6 (C_3), 42.8 ($C_{1'}$), 40.8 ($NHCH_2$), 39.9 (4-CH_2), 34.0 ($C_{3'}$). MS(ES)$^+$: 609.49 $[M+H]^+$. Exact mass calculated for $C_{40}H_{40}N_4O_2$: 608.31513, found 608.31474.

TableS2. Compilation of yield and **a:b** ratio of diastereoisomeric amides **2.10ab** - **2.15ab**.

Compound	R	Ratio of diastereoisomers (a:b)	Yields
**4a,b**	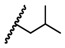	1:1.6	92%
**5a,b**	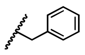	1:2.6	38%
**6a,b**	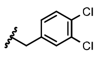	1:1.3	61%
**7a,b**	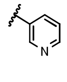	1:1.4	85%
**8a,b**	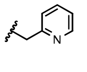	1:1.8	51%
**9a,b**	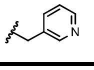	1:1.2	37%

4*R*,*S*-Benzyl-4-methoxycarbonyl-1-[(2'*S*-amino-3'-phenyl)prop-1′-yl]-2-oxoazetidine hydrochloride (10a,b)

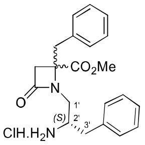

White solid. Yield: 89% (From **2a,b**). Eluent: 2% to 9% of MeOH in DCM. HPLC: t_R = 4.63 min (gradient from 15% to 95% of A in 10 min). Ratio of diastereoisomers M:m, 2.5:1. ^{1}H-NMR (300 MHz, $CDCl_3$, major diastereoisomer **10b**, from the mixture): δ 7.38 - 6.94 (m, 20H, Ar), 3.72 (s, 3H, OCH_3), 3.37 (m, 1H, $H_{2'}$), 3.35 (d, J = 13.8 Hz, 1H, 4-CH_2), 3.28 - 3.14 (m, 3H, $H_{1'}$, H_3), 3.13 (d, J = 13.8 Hz, 1H, 4-CH_2), 2.92 (d, J = 14.8 Hz, 1H, H_3), 2.75 (dd, J = 13.5, 5.3 Hz, 1H, $H_{3'}$), 2.54 (dd, J = 13.5, 8.2 Hz, 1H, $H_{3'}$), 2.36 (s ancho, 3H, NH_3^+). ^{13}C-NMR (75 MHz, $CDCl_3$): δ 172.0 (COO), 167.0 (C_2), 138.6, 134.6, 129.8, 129.4, 128.9, 128.7, 127.6, 126.7 (Ar), 62.8 (C_4), 52.8 (OCH_3), 51.6 ($C_{2'}$), 49.5 ($C_{1'}$), 45.61 (C_3), 42.2 ($C_{3'}$), 39.5 (4-CH_2). ^{1}H-NMR (300 MHz, $CDCl_3$, minor diastereoisomer **10a**, from the mixture): δ 7.38 - 6.97 (m, 10H, Ar), 3.72 (s, 3H, OCH_3), 3.40 - 3.33 (m, 1H, $H_{2'}$), 3.28 - 3.14 (m, 3H, $H_{1'}$, H_3), 2.92 (d, J = 14.8 Hz, 1H, H_3), 2.56 - 252 (m, 2H, $H_{3'}$, 4-CH_2), 2.08 (d, J = 13.9 Hz, 1H, 4-CH_2), 2.05 (dd, J = 11.4, 2.6 Hz, 1H, $H_{3'}$), 2.36 (s ancho, 3H, NH_3^+).^{13}C-NMR (75 MHz, $CDCl_3$): δ 171.3 (COO), 169.5 (C_2), 135.6, 135.5, 130.2, 129.0, 129.1, 129.1, 127.7, 127.3 (Ar), 65.7 (C_4), 52.8 (OCH_3), 51.0 ($C_{2'}$), 48.6 ($C_{1'}$), 45.61 (C_3), 41.9 ($C_{3'}$), 40.2 (4-CH_2). MS(ES)$^+$: 353.18 [M+H]$^+$, 705.57 [2M+H]$^+$.

(3*S*,6*S*)-3,6-Dibenzyl-1,4-diazabiciylo[4.2.0]octane-5,8-dione (11a)

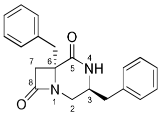

Syrup. Yield: 10% (From **2a,b**). HPLC: t_R = 7.23 min (gradient from 15% to 95% of A in 10 min). ^{1}H-NMR (400 MHz, $CDCl_3$): δ 7.74 - 6.65 (m, 10H, Ar), 5.68 (s ancho, 1H, NH), 3.85 (m, 1H, H_3), 3.70 (dt, J = 14.1, 4.9 Hz, 1H, H_7), 3.59 (dd, J = 13.4, 4.5 Hz, 1H, 3-CH_2), 3.15 (dd, J = 15.6, 4.7 Hz, 1H, H_2), 3.09 (dd, J = 15.8, 4.6 Hz, 1H, H_2), 2.92 (dd, J = 13.4, 4.4 Hz, 1H, 3-CH_2), 2.55 (dt, J = 13.7, 4.7 Hz, 1H, 6-CH_2), 2.07 (m, 2H, 6-CH_2, H_7). ^{13}C-NMR (75 MHz, $CDCl_3$): δ 171.3 (C_5), 169.5 (C_8), 135.5, 134.7, 130.2, 129.4, 129.1, 128.7, 127.7, 127.6 (Ar), 57.9 (C_6), 54.1 (C_3), 48.6 (C_2), 42.0 (C_7), 41.9 (3-CH_2), 40.3 (6-CH_2). MS(ES)$^+$: 321.21 [M+H]$^+$ y 641.49 [2M+H]$^+$. Exact mass calculated for $C_{20}H_{20}N_2O_2$: 320.15248, found 320.15349.

4*R*,*S*-Benzyl-4-[*N*-(*iso*-butyl)carbamoyl]-1-[(2′*S*-amino-3′-phenyl)prop-1′-yl]-2-oxoazetidine hydrochloride (12a,b)

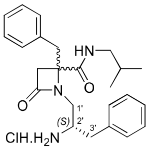

White solid. Yield: 97% (From **4a,b**). HPLC: t_R = 5.18 (m, **12b**) y 5.32 (M, **12a**) min (gradient from 15% to 95% of A in 10 min). Ratio of diastereoisomers M:m, 1.7:1. ^{1}H-NMR (300 MHz, $CDCl_3$, major diastereoisomer**12b**, from the mixture): δ 8.39 (t, J = 5.7 Hz, 1H, 4-CONH), 8.23 (s, 3H, NH_3^+), 7.39 - 7.16 (m, 10H), 3.71 (s ancho, 1H, $H_{2'}$), 3.50 (dd, J = 15.1, 8.6 Hz, 1H, $H_{1'}$), 3.41 (d, J = 14.2 Hz, 1H, 4-CH_2), 3.36 (m, 1H, $H_{1'}$), 3.23 (d, J = 14.1 Hz, 1H, 4-CH_2,), 3.16 (d, J = 14.7 Hz, 1H,

H_3), 3.07 (d, J = 15.1 Hz, 1H, H_3), 3.00 - 2.80 (m, 4H, CH_2, ^{i}Bu, $H_{3'}$), 1.73 (m, 1H, CH, ^{i}Bu), 0.80 (d, J = 6.6 Hz, 3H, CH_3, ^{i}Bu), 0.77 (d, J = 6.7 Hz, CH_3, ^{i}Bu). ^{13}C-NMR (101 MHz, DMSO-d_6): δ 171.2 (4-CONH), 167.8 (C_2), 136.6, 135.6, 130.5, 130.4, 129.8, 129.2, 128.8, 127.4 (Ar), 64.7 (C_4), 51.6, ($C_{2'}$), 47.1 (CH_2, ^{i}Bu), 45.6 (C_3), 42.4 ($C_{1'}$), 39.0 (4-CH_2), 36.7 ($C_{3'}$), 28.2 (CH, ^{i}Bu), 20.7 (CH_3, ^{i}Bu). ^{1}H-NMR (300 MHz, $CDCl_3$, minor diastereoisomer **12a**, from the mixture): δ 8.51 (t, J = 5.8 Hz, 1H, 4-CONH), 8.33 (s, 3H, NH_3^+), 7.39 - 7.16 (m, 10H, Ar), 3.64 (s ancho, 1H, $H_{2'}$), 3.42 (m, 2H, $H_{1'}$), 3.41 (d, J = 14.2 Hz, 1H, 4-CH_2), 3.23 (d, J = 14.1 Hz, 1H, 4-CH_2), 3.16 (d, J = 14.7 Hz, 1H, H_3), 3.07 (d, J = 15.1 Hz, 1H, H_3), 3.00 – 2.80 (m, 4H, CH_2, ^{i}Bu, $H_{3'}$), 1.73 (m, 1H, CH, ^{i}Bu), 0.82 (d, J = 7.4 Hz, 6H, CH_3, ^{i}Bu). ^{13}C-NMR (101 MHz, DMSO-d_6): δ 172.3 (4-CONH), 167.2 (C_2), 136.6, 135.5, 130.5, 130.4, 129.7, 129.2, 128.8, 127.5 (Ar), 64.2 (C_4), 51.8 ($C_{2'}$), 47.1 (CH_2, ^{i}Bu),45.6 (C_3), 43.3 ($C_{1'}$), 38.9 (4-CH_2), 36.9 ($C_{3'}$), 28.2 (CH, ^{i}Bu), 20.7 (CH_3, ^{i}Bu). MS(ES)$^+$: 394.11 [M+H]$^+$.

4*R*,*S*-Benzyl-4-methoxycarbonyl-1-[(2'S-benzylamino-3'-phenyl)prop-1’-yl]-2-oxoazetidine (13a,b)

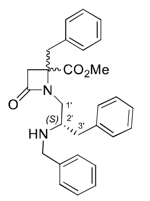

Syrup. Yield: 40% (From **10a,b** and benzaldehyde). Eluent: 14% to 33% of EtOAc in Hexane . HPLC: t_R = 5.61 (M, **13b**) y 5.82 (m, **13a**) min (gradient from 15% to 95% of A in 10 min). Ratio of diastereoisomers M:m, 3.6:1. ^{1}H-NMR (400 MHz, $CDCl_3$, major diastereoisomer): δ 7.45 - 6.95 (m, 15H, Ar), 5.60 (s, 1H, NH), 3.83 (d, J = 13.5 Hz, 1H, NHCH_2), 3.77 (d, J = 13.3 Hz, 1H, NHCH_2), 3.55 (s, 3H, OCH_3), 3.38 (dd, J = 14.0, 6.7 Hz, 1H, $H_{1'}$), 3.28 (d, J = 13.8 Hz, 1H, 4-CH_2), 3.20 (d, J = 14.9 Hz, 1H, H_3),3.13 (m, 2H, $H_{1'}$, $H_{2'}$), 3.01 (d, J = 14.0 Hz, 1H, 4-CH_2), 2.89 (d, J = 14.9 Hz, 1H, H_3), 2.80 (m, 2H, $H_{3'}$). ^{13}C-NMR (75 MHz, $CDCl_3$, major diastereoisomer): δ 171.4 (COO), 167.3 (C_2), 140.3, 138.7, 138.1, 129.7, 129,4, 129.0, 128.8, 128.6, 128.4, 127.8, 127.5, 126.6 (Ar), 63.0 (C_4), 57.7 ($C_{2'}$), 52.5 (OCH_3), 51.4 ($NHCH_2$), 45.8 ($C_{1'}$), 45.3 (C_3), 39.8 (4-CH_2), 39.0 ($C_{3'}$). MS(ES)$^+$: 443.34 [M+H]$^+$. Exact mass calculated for $C_{28}H_{30}N_2O_3$: 442.2256, found 442.2265.

4*R*,*S*-Benzyl-4-methoxycarbonyl-1-[(2'*S*-(3’’-methylbenzyl)amino-3'-phenyl)prop-1’-yl]-2-oxoazetidine (14a,b)

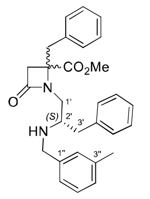

Syrup. Yield: 30% (From **10a,b** and m-tolualdehyde). Eluent: 5% to 100% of EtOAc in DCM. HPLC: t_R = 6.13 (M, **14b**) y 7.27 (m, **14a**) min (gradient from 15% to 95% of A in 10 min). Ratio of diastereoisomers M:m, 3.6:1. ^{1}H-NMR (400 MHz, $CDCl_3$, major diastereoisomer): δ 7.45 - 6.80 (m, 14H, Ar), 3.75 (s, 2H, NHCH_2), 3.57 (s, 3H, OCH_3), 3.37 (dd, J = 14.2, 6.7 Hz, 1H, $H_{1'}$), 3.31 (d, J = 14.0 Hz, 1H, 4-CH_2), 3.22 (d, J = 15.0 Hz, 1H, H_3), 3.20 (m, 1H, $H_{1'}$), 3.12 (m, 1H, $H_{2'}$), 3.00 (d, J = 13.9 Hz, 1H, 4-CH_2), 2.90 (d, J = 14.8 Hz, 1H, H_3), 2.78 (m, 2H, $H_{3'}$), 2.30 (s, 3H, 3’’- CH_3), 1.94 (s, 1H, NH). ^{13}C-NMR (75 MHz, $CDCl_3$, major diastereoisomer): δ 171.4 (COO), 167.2 (C_2), 140.3, 138.7, 138.1, 134.7, 129.7, 129,4, 129.0, 128.8, 128.6, 128.4, 127.8, 127.5, 126.5, 125.3 (Ar), 63.0 (C_4), 57.6 ($C_{2'}$), 52.51 (OCH_3), 51.4 ($NHCH_2$), 45.8 ($C_{1'}$), 45.3 (C_3), 39.8 (4-CH_2), 39.4 ($C_{3'}$), 21.5 (3’’-

CH_3). MS(ES)$^+$: 457.16 $[M+H]^+$. Exact mass calculated for $C_{29}H_{32}N_2O_3$: 456.24129, found 456.24161.

4*R*,*S*-Benzyl-4-methoxycarbonyl-1-[(2'*S* -(4''-methylbenzyl)amino-3'- phenyl)prop-1'-yl]-2-oxoazetidine (15a,b)

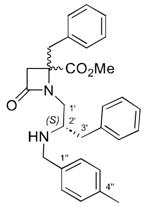

Syrup. Yield: 30% (From **2.16ab** and p-tolualdehyde). Eluent: 17% to 100% of EtOAc in DCM. HPLC: t_R = 5.40 (M, **15b**) y 6.50 (m, **15a**) min (gradient from 20% to 95% of A in 10 min). Ratio of diastereoisomers M:m, 5.6:1. ^{1}H-NMR (400 MHz, $CDCl_3$, major diastereoisomer): δ 7.35 - 6.92 (m, 14H, Ar), 3.75 (d, J = 13.1 Hz, 1H, NHCH_2), 3.72 (d, J = 13.2 Hz, 1H, NHCH_2), 3.55 (s, 3H, OCH_3), 3.33 (dd, J = 14.2, 6.7 Hz, 1H, $H_{1'}$), 3.30 (d, J = 14.0 Hz, 1H, 4-CH_2), 3.21 (d, J = 15.0 Hz, 1H, H_3), 3.19 (m, 1H, $H_{2'}$), 3.11 (m, 1H, $H_{1'}$), 3.00 (d, J = 13.9 Hz, 1H, 4-CH_2), 2.89 (dd, J = 14.3, 4.3 Hz, 1H, $H_{1'}$), 2.89 (d, J = 14.8 Hz, 1H, H_3), 2.78 (m, 2H, $H_{3'}$), 2.30 (s, 3H, 4''-CH_3), 2.17 (s, 1H, NH). ^{13}C-NMR (75 MHz, $CDCl_3$, major diastereoisomer): δ 171.4 (COO), 167.2 (C_2), 138.7, 137.2, 136.5, 134.7, 129.7, 129.4, 129.1, 128.8, 128.6, 128.2, 127.5, 126.5 (Ar), 62.7 (C_4), 57.6 ($C_{2'}$), 52.5 (OCH_3), 51.2 ($NHCH_2$), 45.8 ($C_{1'}$), 45.3 (C_3), 39.7 (4-CH_2), 39.4 ($C_{3'}$), 21.2 (4''-CH_3). MS(ES)$^+$: 457.16 $[M+H]^+$. Exact mass calculated for $C_{29}H_{32}N_2O_3$: 456.24129, found 456.24234.

4*R*,*S*-Benzyl-4-methoxycarbonyl-1-[(2'*S*-(3''-bromobenzyl)amino-3'-phenyl)prop-1'-yl]-2-oxoazetidine (16a,b)

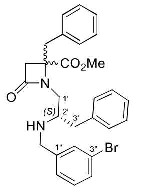

Syrup. Yield: 40% (From **10a,b** and 3-bromobenzaldehyde). Eluent: 17% to 100% of EtOAc in DCM. HPLC: t_R = 6.83 (M, **16b**) y 7.82 (m, **16a**) min (gradient from 10% to 95% of A in 10 min). Ratio of diastereoisomers M:m, 2:1. ^{1}H-NMR (400 MHz, $CDCl_3$, major diastereoisomer, from the mixture): δ 7.43 - 6.93 (m, 14H, Ar), 3.76 (d, J = 13.7 Hz, 1H, NHCH_2), 3.71 (d, J = 13.8 Hz, 1H, NHCH_2), 3.58 (s, 3H, OCH_3), 3.34 (dd, J = 14.2, 7.1 Hz, 1H, $H_{1'}$), 3.28 (d, J = 14.0 Hz, 1H, 4-CH_2), 3.24 (m, 1H, $H_{1'}$), 3.16 (d, J = 15.1 Hz, 1H, H_3), 3.16 (m, 1H, $H_{2'}$), 3.02 (d, J = 13.9 Hz, 1H, 4-CH_2), 2.91 (d, J = 14.8 Hz, 1H, H_3), 2.76 (m, 2H, $H_{3'}$) 1.87 (s, 1H, NH). ^{13}C-NMR (75 MHz, $CDCl_3$, major diastereoisomer): δ 171.5 (COO), 167.2 (C_2), 143.0, 138.6, 134.5, 131.3, 130.1, 130.0, 129.7, 129.4, 128.9, 128.7, 127.6, 126.8, 126.6, 122.6 (Ar), 63.0 (C_4), 57.6 ($C_{2'}$), 52.6 (OCH_3), 50.7 ($NHCH_2$), 45.9 ($C_{1'}$), 45.4 (C_3), 39.8 (4-CH_2), 39.5 ($C_{3'}$). MS(ES)$^+$: 521.30 $[M+H]^+$. Exact mass calculated for $C_{28}H_{29}BrN_2O_3$: 520.13616, found 520.13646. ^{1}H-NMR (400 MHz, $CDCl_3$, minor diastereoisomer, from the mixture): δ 7.39 - 6.95 (m, 14H, Ar), 3.78-3.68 (m, *2*H, NHCH_2), 3.63 (s, 3H, OCH_3), 3.38 (m, 1H, $H_{1'}$), 3.26-2.68 (m, 8H, 4-CH_2, $H_{1'}$, H_3, $H_{2'}$, H_3), 2.53 (m, 2H, $H_{3'}$) 1.87 (s, 1H, NH). ^{13}C-RMN (75 MHz, $CDCl_3$: δ 171.6 (COO), 167.1 (C_2), 143.1, 138.6, 134.7, 131.3, 130.1, 130.0, 129.7, 129.4, 129.0, 128.6, 127.6, 126.9, 126.7, 122.6 (Ar), 63.1 (C_4), 57.4 ($C_{2'}$), 52.6 (OCH_3), 50.8 ($NHCH_2$), 46.1 ($C_{1'}$), 45.6 (C_3), 39.9 (4-CH_2), 39.6 ($C_{3'}$) .

4*R*,*S*-Benzyl-4-methoxycarbonyl-1-[(2'*S*-naphtylmethylamino-3'-phenyl)prop-1'-yl]-2-oxoazetidine (17a,b)

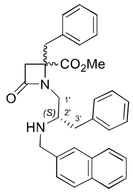

Syrup. Yield: 45% (From **10a,b** and 2-naphtaldehyde). Eluent: 1% to 10% of MeOH in DCM. HPLC: t_R = 6.47 (M, **17b**) y 7.21 (m,**17a**) min (gradient from 15% to 95% of A in 10 min). Ratio of diastereoisomers M:m, 4:1. ^{1}H-NMR (400 MHz, $CDCl_3$, major diastereoisomer): δ 7.92 - 6.78 (m, 17H, Ar), 3.97 (d, J = 13.6 Hz, 1H, NHCH_2), 3.91 (d, J = 13.7 Hz, 1H, NHCH_2), 3.44 (s, 3H, OCH_3), 3.39 (dd, J = 13.9, 6.6 Hz, 1H, $H_{1'}$), 3.27 (d, J = 13.9 Hz, 1H, 4-CH_2), 3.25 (m, 1H, $H_{1'}$), 3.21 (d, J = 14.7 Hz, 1H, H_3), 3.15 (m, 1H, $H_{2'}$), 2.98 (d, J = 14.0 Hz, 1H, 4-CH_2), 2.89 (d, J = 14.8 Hz, 1H, H_3), 2.80 (m, 2H, $H_{3'}$), 1.78 (s, 1H, NH). ^{13}C-NMR (75 MHz, $CDCl_3$, major diastereoisomer): δ 171.4 (COO), 167.3 (C_2), 138.8, 134.6, 132.8, 130.5, 129.7, 129.4, 128.8, 128.7, 128.7, 128.1, 127.8, 127.7, 127.5, 126.7, 126.6, 126.6, 126.1, 125.6 (Ar), 63.0 (C_4), 57.6 ($C_{2'}$), 52.4 (OCH_3), 51.5 ($NHCH_2$), 45.9 ($C_{1'}$), 45.3 (C_3), 39.8 (4-CH_2), 39.5 ($C_{3'}$). MS(ES)$^+$: 493.44 [M+H]$^+$. Exact mass calculated for $C_{32}H_{32}N_2O_3$: 492.24129, found 492.24262.

4*R*,*S*-Benzyl-4-methoxycarbonyl-1-[(2'*S*-(4''-propoxybenzyl)amino-3'-phenyl) prop-1'-yl]-2-oxoazetidine (18a,b)

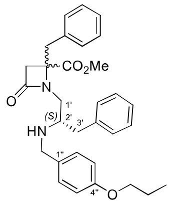

Syrup. Yield: 36% (From **10a,b** and 4-propoxybenzaldehyde). Eluent: 17% to 100% of EtOAc in DCM. HPLC: t_R = 6.07 (M, **18b**) y 6.54 (m, **19a**) min (gradient from 20% to 95% of A in 10 min). Ratio of diastereoisomers M:m, 9.5:1. ^{1}H-NMR (400 MHz, $CDCl_3$, major diastereoisomer): δ 7.39 - 6.66 (m, 14H, Ar), 3.88 (t, J = 6.6 Hz, 2H, OCH_2, Pr), 3.73 (d, J = 13.3 Hz, 1H, NHCH_2), 3.69 (d, J = 13.4 Hz, 1H, NHCH_2), 3.57 (s, 3H, OCH_3), 3.36 (dd, J = 14.0, 6.7 Hz, 1H, $H_{1'}$), 3.30 (d, J = 13.9 Hz, 1H, 4-CH_2), 3.21 (d, J = 15.0 Hz, 1H, H_3), 3.20 (m, 1H, $H_{1'}$), 3.13 (m, 1H, $H_{2'}$), 3.00 (d, = 14.0 Hz, 1H, 4-CH_2), 2.89 (d, J = 14.8 Hz,1H, H_3), 2.77 (m, 2H, $H_{3'}$), 1.88 (s, 1H, NH), 1.77 (m, 2H, CH_2, Pr), 1.02 (t, J = 7.4 Hz, 3H, CH_3, Pr). ^{13}C-NMR (75 MHz, $CDCl_3$, major diastereoisomer): δ 171.5 (COO), 167.2 (C_2), 158.3, 138.8, 134.7, 132.2, 129.7, 129.4, 129.4, 128.8, 128.6, 127.5, 126.5, 114.5 (Ar), 69.7 (OCH_2, Pr), 63.0 (C_4), 57.6 ($C_{2'}$), 52.5 (OCH_3), 50.9 ($NHCH_2$), 45.9 ($C_{1'}$), 45.3 (C_3), 39.8 (4-CH_2), 39.4 ($C_{3'}$), 22.7 (CH_2, Pr), 10.7 (CH_3, Pr). MS(ES)$^+$: 501.32 [M+H]$^+$. Exact mass calculated for $C_{31}H_{36}N_2O_4$: 500.26751, found 500.26864.

4*R*,*S*-Benzyl-4-[*N*-(*iso*-butyl)carbamoyl]-1-[(2'*S*-(3''-methylbenzyl)amino-3'- phenyl)prop-1'-yl] -2-oxoazetidine (19a,b)

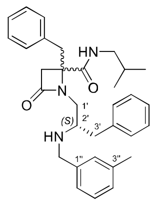

Syrup. Yield: 28% (From **12a,b** and 3-tolualdehyde). Eluent: 6% to 50% of EtOAc in DCM. HPLC: t_R = 6.49 (m, **19a**) y 6.77 (M, **19b**) min (gradient from 15% to 95% of A in 10 min). Ratio of diastereoisomers M:m, 1.5:1. ^{1}H-NMR (300 MHz, $CDCl_3$, major diastereoisomer **19b**, from the mixture): δ 10.14 (s, 1H, 4-CONH), 7.36 - 6.54 (m, 15H, Ar, 2'-NH), 3.94 (d, J = 14.4 Hz, 1H, 4-CH_2), 3.61 (dd, J = 15.0, 6.3 Hz, 1H, $H_{1'}$), 3.41 (d, J = 12.8 Hz, 1H, NHCH_2,), 3.34 (d, J = 12.8 Hz, 1H, NHCH_2), 3.16 - 2.95 (m, 5H, CH_2, ^{i}Bu, $H_{1'}$, H_3, 4-CH_2), 2.65 (m, 2H, $H_{2'}$, $H_{3'}$), 2.61 - 2.49 (m, 2H, $H_{3'}$, CH_2, ^{i}Bu), 2.24 (s, 3H, 3''-CH_3), 1.57 (m, 1H, CH, ^{i}Bu), 0.75 (d, J = 6.2 Hz, 3H, CH_3, ^{i}Bu), 0.73 (d, J = 6.3 Hz, 3H, CH_3, ^{i}Bu). ^{13}C-NMR (75 MHz, $CDCl_3$): δ 172.6 (4-CONH), 169.5 (C_2), 138.3, 137.9, 136.2, 130.2, 129.5, 129.1, 128.9, 128.7, 128.6, 128.2, 127.4, 126.9, 125.1 (Ar), 65.2 (C_4), 56.4 ($C_{2'}$), 50.1 (C_3), 48.1 (NHCH_2), 46.9 (CH_2, ^{i}Bu), 43.2 ($C_{1'}$), 40.5 (4-CH_2), 38.4 ($C_{3'}$), 27.8 (CH, ^{i}Bu), 21.4 (3''-CH_3), 20.3, 20.2 (CH_3, ^{i}Bu). ^{1}H-NMR (300 MHz, $CDCl_3$, minor diastereoisomer **19a**, from the mixture): δ 9.63 (s, 1H, 4-CONH), 7.36 - 6.54 (m, 15H, Ar, 2'-NH), 3.87 (d, J = 13.9 Hz, 1H, 4-CH_2), 3.79 (m, 1H, $H_{2'}$), 3.70 (d, J = 11.8 Hz, 1H, NHCH_2), 3.56 (d, J = 11.9 Hz, 1H, NHCH_2), 3.16 - 2.95 (m, 5H, $H_{1'}$, H_3, CH_2, ^{i}Bu, 4-CH_2), 2.65 (m, 1H, $H_{3'}$), 2.52 (m, 1H, $H_{3'}$), 2.30 (s, 3H, 3''- CH_3), 2.05 - 1.87 (m, 2H, CH_2, ^{i}Bu, $H_{1'}$), 1.24 (m, 1H, CH, ^{i}Bu), 0.49 (d, J = 6.6 Hz, 3H, CH_3, ^{i}Bu), 0.35 (d, J = 6.6 Hz, 3H, CH_3, ^{i}Bu). ^{13}C-NMR (75 MHz, $CDCl_3$): δ 171.5 (4-CONH), 167.7 (C_2), 139.0, 138.5, 136.6, 136.4, 130.3, 129.5, 128.9, 128.8, 128.5, 128.4, 127.2, 127.1, 125.7 (Ar), 64.3 (C_4), 55.1 ($C_{2'}$), 53.1 (NHCH_2), 49.9 (CH_2, ^{i}Bu), 49.6 (C_3), 46.4 ($C_{1'}$), 41.1 (4-CH_2), 40.5 ($C_{3'}$), 27.8 (CH, ^{i}Bu), 21.4 (3''-CH_3), 20.0, 19.9 (CH_3, ^{i}Bu). MS(ES)$^+$: 498.34 [M+H]$^+$. Exact mass calculated for $C_{32}H_{39}N_3O_2$: 497.30423, found 497.30343.

4*R*,*S*-Benzyl-4-[*N*-(*iso*-butyl)carbamoyl]-1-[(2'*S*-(3''-biphenyl)methylamino-3'- phenyl)prop-1'-yl] -2-oxoazetidine (20a,b)

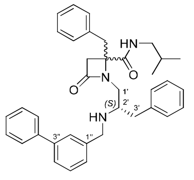

Syrup. Yield: 80% (From **12a,b** and biphenyl-3-carboxaldehyde). Eluent: 6% to 20% of EtOAc in DCM. HPLC: t_R = 7.16 (m, **20a**) y 7.42 (M, **20b**) min (gradient from 15% to 95% of A in 10 min). Ratio of diastereoisomers M:m, 1.1:1. ^{1}H-NMR (300 MHz, $CDCl_3$, major diastereoisomer **20b**, from the mixture): δ 10.11 (t, J = 5.8 Hz, 1H, 4-CONH), 7.57 - 6.74 (m, 20H, Ar, 2'-NH), 3.97 (d, J = 14.3 Hz, 1H, 4-CH_2), 3.65 (dd, J = 14.9, 6.2 Hz, 1H, $H_{1'}$), 3.44 (d, J = 12.8 Hz, 1H, NHCH_2), 3.38 (d, J = 12.8 Hz, 1H, NHCH_2), 3.20 - 2.98 (m, 5H, CH_2, ^{i}Bu, $H_{1'}$, 2H_3, 4-CH_2), 2.83 - 2.67 (m, 2H, $H_{3'}$, $H_{2'}$), 2.65 -2.50 (m, 2H, CH_2, ^{i}Bu, $H_{3'}$), 1.25 (m, 1H, CH, ^{i}Bu), 0.46 (d, J = 6.7 Hz, 3H, CH_3, ^{i}Bu), 0.33 (d, J = 6.6 Hz, 3H, CH_3, ^{i}Bu). ^{13}C-NMR (75 MHz, $CDCl_3$): δ 172.5 (4-CONH), 169.4 (C_2), 141.7, 140.7, 138.9, 137.8, 136.1, 130.1, 129.1, 129.0, 128.8, 128.6, 127.5, 127.2, 127.1, 126.9, 126.8, 126.3 (Ar), 65.2 (C_4), 56.4 ($C_{2'}$), 50.1 (C_3), 48.1 (NHCH_2), 46.9 (CH_2, ^{i}Bu), 43.1 ($C_{1'}$), 40.5 (4-CH_2), 38.3 ($C_{3'}$), 28.2 (CH, ^{i}Bu), 20.6, 20.3 (CH_3, ^{i}Bu). ^{1}H-NMR (300 MHz, $CDCl_3$, minor diastereoisomer **20a**, from the mixture): δ 9.60 (t, J = 5.6 Hz, 1H, 4-CONH), 7.57 - 6.74 (m, 20H, Ar, 2'-NH), 3.88 (m, 2H, 4-CH_2, $H_{2'}$), 3.80 (d, J = 11.9 Hz, 1H, NHCH_2), 3.68 (d, J = 12.0 Hz, 1H, NHCH_2), 3.21 (dd, J =

14.1, 3.0 Hz, 1H, $H_{1'}$), 3.20 - 2.98 (m, 4H, H_3, CH_2, ^{i}Bu, 4-CH_2), 2.70 (m, 1H, $H_{3'}$), 2.55 (m, 1H, $H_{3'}$), 2.03 (m, 1H, $H_{1'}$), 1.94 (m, 1H, CH_2, ^{i}Bu), 1.56 (m, 1H, CH, ^{i}Bu), 0.71 (t app, J = 6.8 Hz, 6H, CH_3, ^{i}Bu). ^{13}C-NMR (75 MHz, $CDCl_3$): δ 171.4 (4-CONH), 167.7 (C_2), 141.9, 140.8, 139.6, 136.6, 136.3, 130.2, 129.4, 129.3, 128.9, 128.6, 127.6, 127.4, 127.1, 127.1, 126.9, 126.4 (Ar), 64.3 (C_4), 55.1 ($C_{2'}$), 53.1($NHCH_2$), 49.8 (CH_2, ^{i}Bu), 49.5 (C_3), 46.4 ($C_{1'}$), 41.0 (4-CH_2), 38.5 ($C_{3'}$), 27.8 (CH, ^{i}Bu), 20.0, 19.8 (CH_3, ^{i}Bu). MS(ES)$^+$: 560.49 [M+H]$^+$. Exact mass calculated for $C_{37}H_{41}N_3O_2$: 559.31988, found 559.32211.

4*R*,*S*-Benzyl-4-[*N-(iso*-butyl)carbamoyl]-1-[(2'*S*-(3''-fluorobenzyl)amino-3'- phenyl)prop-1'-yl]-2-oxoazetidine (21a,b)

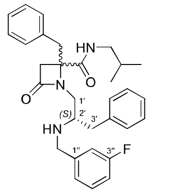

Syrup. Yield: 32% (From **12a,b** and 3-fluorobenzaldehyde). Eluent: 9% to 33% of EtOAc in DCM. HPLC: t_R = 6.49 (m, **21a**) y 6.72 (M, **21b**) min (gradient from 15% to 95% of A in 10 min). Ratio of diastereoisomers M:m, 2.6:1. ^{1}H-NMR (300 MHz, $CDCl_3$, major diastereoisomer **21b**, from the mixture): δ 9.80 (s, 1H, 4-CONH), 7.39 - 6.45 (m, 20H, Ar, 2'-NH), 3.92 (d, J = 14.3 Hz, 1H, 4-CH_2), 3.62 (m, 1H, $H_{1'}$), 3.39 (d, J = 13.1 Hz, 1H, $NHCH_2$), 3.35 (d, J = 13.2 Hz, 1H, $NHCH_2$), 3.20 - 2.98 (m, 5H, CH_2, ^{i}Bu, $H_{1'}$, H_3, 4-CH_2), 2.75 - 2.50 (m, 4H, CH_2, ^{i}Bu, $H_{2'}$, $H_{3'}$), 1.57 (m, 1H, CH, ^{i}Bu), 0.74 (d, J = 6.7 Hz, 3H, CH_3, ^{i}Bu), 0.73 (d, J = 6.6 Hz, 3H, CH_3, ^{i}Bu). ^{13}C-NMR (75 MHz, $CDCl_3$): δ 172.6 (4-CONH), 169.4 (C_2), 162.9 (d, J = 246.8 Hz, $C_{3''}$), 140.9 (d, J = 6.7 Hz, $C_{1''}$), 137.6, 136.5, 130.5 (d, J = 8.2 Hz, $C_{5''}$), 130.1, 129.0, 129.0, 128.9, 127.5, 127.1, 123.6 (d, J = 2.8 Hz, $C_{6''}$), 114.9 (d, J = 21.4 Hz, $C_{2''}$), 114.4 (d, J = 21.3 Hz, $C_{4''}$) (Ar), 65.2 (C_4), 56.3 ($C_{2'}$), 50.2 (C_3), 47.4 ($NHCH_2$), 47.0 (CH_2, ^{i}Bu), 43.1 ($C_{1'}$), 40.4 (4-CH_2), 38.2 ($C_{3'}$), 28.3 (CH, ^{i}Bu), 20.3, 20.2 (CH_3, ^{i}Bu). ^{1}H-NMR (300 MHz, $CDCl_3$, minor diastereoisomer **21a**, from the mixture): δ 9.38 (s, 1H, 4-CONH), 7.39 - 6.75 (m, 20H, Ar, 2'-NH), 3.85 (d, J = 13.9 Hz, 4-CH_2), 3.76 (m, 1H, $H_{2'}$), 3.69 (d, J = 12.2 Hz, 1H, $NHCH_2$), 3.62 (m, 1H, $NHCH_2$), 3.25 (dd, J = 13.9, 2.6 Hz, 1H, $H_{1'}$), 3.20 - 2.98 (m, 4H, H_3, CH_2, ^{i}Bu, 4-CH_2), 2.75 - 2.50 (m, 2H, $H_{3'}$), 2.12 - 1.99 (m, 2H, $H_{1'}$, CH_2, ^{i}Bu), 1.28 (m, 1H, CH, ^{i}Bu), 0.53 (d, J = 6.7 Hz, 3H, CH_3, ^{i}Bu), 0.42 (d, J = 6.6 Hz, 3H, CH_3, ^{i}Bu). ^{13}C-NMR (75 MHz, $CDCl_3$): δ 171.5 (4-CONH), 167.8 (C_2), 163.0 (d, J = 246.8 Hz, $C_{3''}$), 141.4 (d, J = 8.0 Hz, $C_{1''}$), 136.2, 136.0, 130.4 (d, J = 7.6 Hz, $C_{5''}$), 130.3, 130.2, 129.4, 128.7, 127.3, 127.2, 124.4 (d, J = 2.8 Hz, $C_{6''}$), 115.6 (d, J = 21.0 Hz, $C_{2''}$), 114.6 (d, J = 21.7 Hz, $C_{4''}$) (Ar), 64.3 (C_4), 55.1 ($C_{2'}$), 52.1 ($NHCH_2$), 49.7 (CH_2, ^{i}Bu), 49.5 (C_3), 46.6 ($C_{1'}$), 41.0 (4-CH_2), 38.6 ($C_{3'}$), 28.0 (CH, ^{i}Bu), 20.1, 19.9 (CH_3, ^{i}Bu). MS(ES)$^+$: 502.39 [M+H]$^+$. Exact mass calculated for $C_{31}H_{36}FN_3O_2$: 501.27916, found 501.27949.

4*R*,*S*-Benzyl-4-[*N-(iso*-butyl)carbamoyl]-1-[(2'*S*-(3''-chlorobenzyl)amino-3'- phenyl)prop-1'-yl]-2-oxoazetidine (22a,b)

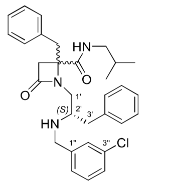

Syrup. Yield: 27% (From **12a,b** and 3-chlorobenzaldehyde). Eluent: 9% to 33% of EtOAc in DCM. HPLC: t_R = 6.77 (m, **22a**) y 7.02 (M, **22b**) min (gradient from 15% to 95% of A in 10 min). Ratio of

diastereoisomers M:m, 2.4:1. ^{1}H-NMR (300 MHz, $CDCl_3$, major diastereoisomer **22 b**, from the mixture): δ 9.78 (t, J = 5.9 Hz, 1H, 4-CONH), 7.28 - 6.66 (m, 20H, Ar, 2'-NH), 3.94 (d, J = 14.3 Hz, 1H, 4-CH_2), 3.37 (d, J = 13.2 Hz, 1H, NHCH_2), 3.31 (d, J = 13.3 Hz, 1H, NHCH_2), 3.13 - 2.88 (m, 5H, CH_2, ^{i}Bu, $H_{1'}$, 4-CH_2, H_3), 2.77 -2.50 (m, 2H, $H_{3'}$), 2.18 - 1.99 (m, 2H, $H_{1'}$, CH_2, ^{i}Bu), 1.57 (m, 1H, CH, ^{i}Bu), 0.76 (d, J = 6.6 Hz, 3H, CH_3, ^{i}Bu), 0.73 (d, J = 6.6 Hz, 3H, CH_3, ^{i}Bu). ^{13}C-NMR (75 MHz, $CDCl_3$): δ 172.5 (4-CONH), 169.4 (C_2), 140.4, 137.6, 136.5, 134.5, 130.1, 129.9, 129.0, 128.9, 128.9, 128.2, 127.7, 127.5, 127.1, 126.2 (Ar), 65.2 (C_4), 56.2 ($C_{2'}$), 50.2 (C_3), 47.4 (NHCH_2), 47.0 (CH_2, ^{i}Bu), 43.0 ($C_{1'}$), 40.5 (4-CH_2), 38.2 ($C_{3'}$), 28.3 (CH, ^{i}Bu), 20.32, 20.25 (CH_3, ^{i}Bu). ^{1}H-NMR (300 MHz, $CDCl_3$, major diastereoisomer **22a**, from the mixture): δ 9.34 (s ancho, 1H, 4-CONH), 7.28 - 6.88 (m, 20H, Ar, 2'-NH), 3.86 (d, J = 14.0 Hz, 4-CH_2), 3.76 (m, 1H, $H_{2'}$), 3.68 (d, J = 12.3 Hz, 1H, NHCH_2), 3.60 (m, 2H, $H_{1'}$, NHCH_2), 3.18 (dd, J = 13.5, 6.9 Hz, 1H, $H_{1'}$), 3.13 - 2.88 (m, 4H, H_3, CH_2, ^{i}Bu, 4-CH_2), 2.77 -2.50 (m, 2H, $H_{3'}$), 2.18 - 1.99 (m, 2H, $H_{1'}$, CH_2, ^{i}Bu), 1.27 (m, 1H, CH, ^{i}Bu), 0.54 (d, J = 6.7 Hz, 3H, CH_3, ^{i}Bu), 0.43 (d, J = 6.6 Hz, 3H, CH_3, ^{i}Bu). ^{13}C-NMR (75 MHz, $CDCl_3$): δ 171.5 (4-CONH), 167.7 (C_2), 141.0, 136.3, 136.0, 134.7, 130.2, 129.4, 129.2, 128.9, 128.6, 127.9, 127.3, 127.2, 126.9, 124.0 (Ar), 64.3 (C_4), 55.1 ($C_{2'}$), 52.4 (NHCH_2), 49.8 (CH_2, ^{i}Bu), 49.5 (C_3), 46.6 ($C_{1'}$), 41.0 (4-CH_2), 38.6 ($C_{3'}$), 28.0 (CH, ^{i}Bu), 20.1, 19.9 (CH_3, ^{i}Bu). MS(ES)$^+$: 518.24 [M+H]$^+$. Exact mass calculated for $C_{31}H_{36}ClN_3O_2$: 517.24961, found 517.2505.

4*R*,*S*-Benzyl-4-[*N-(iso*-butyl)carbamoyl]-1-[(2'*S*-(3''-bromobenzyl)amino-3'- phenyl) prop-1'-yl]-2-oxoazetidine (23a,b)

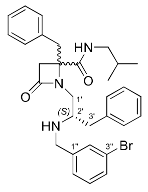

Syrup. Yield: 30% (From **12a,b** and 3-bromobenzaldehyde). Eluent: 9% to 16% of EtOAc in DCM. HPLC: t_R = 6.86 (m, **23a**) y 7.13 (M, **23b**) min (gradient from 15% to 95% of A in 10 min). Ratio of diastereoisomers M:m, 2.9:1. ^{1}H-NMR (300 MHz, $CDCl_3$, major diastereoisomer **23b**, from the mixture): δ 9.81 (t, J = 6.0 Hz, 1H, 4-CONH), 7.44 - 6.69 (m, 15H, Ar, 2'-NH), 3.96 (d, J = 14.3 Hz, 1H, 4-CH_2), 3.60 (m, 1H, $H_{1'}$), 3.35 (d, J = 13.2 Hz, 1H, NHCH_2), 3.29 (d, J = 13.2 Hz, 1H, NHCH_2), 3.15 - 2.85 (m, 5H, CH_2, ^{i}Bu, $H_{1'}$, H_3, 4-CH_2), 2.77 - 2.44 (m, 4H, CH_2, ^{i}Bu, $H_{2'}$, $H_{3'}$), 1.59 (m, 1H, CH, ^{i}Bu), 1.27 (m, 1H, CH, ^{i}Bu), 0.76 (d, J = 6.6 Hz, 3H, CH_3, ^{i}Bu), 0.73 (d, J = 6.5 Hz, 3H, CH_3, ^{i}Bu). ^{13}C-NMR (75 MHz, $CDCl_3$): δ 172.5 (4-CONH), 169.4 (C_2), 140.8, 137.6, 136.6, 131.1, 130.6, 130.3, 130.2, 129.1, 128.9, 128.9, 127.5, 127.1, 126.6, 122.7 (Ar), 65.2 (C_4), 56.2 ($C_{2'}$), 50.3 (C_3), 47.3 (NHCH_2), 47.0 (CH_2, ^{i}Bu), 43.0 ($C_{1'}$), 40.5 (4-CH_2), 38.3 ($C_{3'}$), 28.4 (CH, ^{i}Bu), 20.4, 20.3 (CH_3, ^{i}Bu). ^{1}H-NMR (300 MHz, $CDCl_3$, minor diastereoisomer **23a**, from the mixture):δ 9.36 (t, J = 6.6 Hz, 1H, 4-CONH), 7.44 - 6.87 (m, 15H, Ar, 2'-NH),3.87 (d, J = 13.9 Hz, 4-CH_2), 3.77 (m, 1H, $H_{2'}$), 3.67 (d, J = 12.1 Hz, 1H, NHCH_2), 3.60 (m, 1H, NHCH_2), 3.19 (dd, J = 13.3, 6.8 Hz, 1H, $H_{1'}$), 3.15 - 2.85 (m, 4H, CH_2, ^{i}Bu, H_3, 4-CH_2), 2.77 -2.44 (m, 2H, $H_{3'}$), 2.18 - 1.96 (m, 2H, $H_{1'}$, CH_2, ^{i}Bu), 1.27 (m, 1H, CH, ^{i}Bu), 0.54 (d, J = 6.6 Hz, 3H, CH_3, ^{i}Bu), 0.43 (d, J = 6.6 Hz, 3H, CH_3, ^{i}Bu). ^{13}C-NMR (75 MHz, $CDCl_3$): δ 171.4 (4-CONH), 167.8 (C_2), 141.4, 136.3, 136.0, 131.8, 130.8, 130.4, 130.2, 129.4, 129.1, 128.6, 127.4, 127.3, 127.2, 122.9 (Ar), 64.3 (C_4), 55.1 ($C_{2'}$), 52.5 (NHCH_2), 49.9 (CH_2, ^{i}Bu), 49.6 (C_3), 46.6

(C$_{1'}$), 41.0 (4-CH$_2$), 38.7 (C$_{3'}$), 28.0 (CH, ^{i}Bu), 20.1, 20.0 (CH$_3$, ^{i}Bu). MS(ES)$^+$: 562.33 [M+H]$^+$ y 564.24 [M+2]$^+$. Exact mass calculated for $C_{31}H_{36}BrN_3O_2$: 561.19909, found 561.20026.

4*R*,*S*-Benzyl-4-[*N-(iso*-butyl)carbamoyl]-1-[(2'*S*-(3-iodobenzyl)amino-3'- phenyl)prop-1'-yl]-2-oxoazetidine (24a,b)

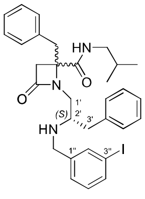

Syrup. Yield: 19% (From **12a,b** and 3-iodobenzaldehyde). Eluent: 9% to 33% of EtOAc in DCMe. HPLC: t$_R$ = 6.91 (m, **24a**) y 7.16 (M, **24b**) min (gradient from 15% to 95% of A in 10 min). Ratio of diastereoisomers M:m, 2.7:1. ^{1}H-NMR (300 MHz, CDCl$_3$, major diastereoisomer **24b**, from the mixture): δ 9.82 (s, 1H, 4-CONH), 7.55 - 6.70 (m, 15H, Ar, 2'-NH), 3.96 (d, *J* = 14.3 Hz, 1H, 4-CH$_2$), 3.32 (d, *J* = 13.1 Hz, 1H, NHC*H*$_2$), 3.25 (d, *J* = 13.1 Hz, 1H, NHC*H*$_2$), 3.12 - 2.88 (m, 5H, CH$_2$, ^{i}Bu, H$_{1'}$, H$_3$, 4-CH$_2$), 2.79 -2.51 (m, 4H, CH$_2$, ^{i}Bu, H$_{2'}$, H$_{3'}$), 1.58 (m, 1H, CH, ^{i}Bu), 0.77 (d, *J* = 6.5 Hz, 3H, CH$_3$, ^{i}Bu), 0.76 (d, *J* = 6.5 Hz, 3H, CH$_3$, ^{i}Bu). ^{13}C-NMR (75 MHz, CDCl$_3$): δ 172.5 (4-CONH), 169.4 (C$_2$), 140.8, 137.6, 137.1, 136.6, 130.4, 130.2, 129.1, 129.0, 128.9, 128.6, 127.5, 127.3, 127.1, 94.6 (Ar), 65.2, (C$_4$), 56.2 (C$_{2'}$), 50.3 (C$_3$), 47.3 (NHCH$_2$), 47.1 (CH$_2$, ^{i}Bu), 43.0 (C$_{1'}$),40.6 (4-CH$_2$), 38.3 (C$_{3'}$), 28.4 (CH, ^{i}Bu), 20.4, 20.3 (CH$_3$, ^{i}Bu). ^{1}H-NMR (300 MHz, CDCl$_3$, minor diastereoisomer **24a**, from the mixture): δ 9.34 (s, 1H, 4-CONH), 7.64 - 6.90 (m, 15H, Ar, 2'-NH), 3.87 (d, *J* = 14.0 Hz, 4-CH$_2$), 3.75 (m, 1H, H$_{2'}$), 3.64 (d, *J* = 11.5 Hz, 1H, NHC*H*$_2$), 3.60 (m, 2H, H$_{1'}$, NHC*H*$_2$), 3.18 (dd, *J* = 13.1, 6.6 Hz, 1H, H$_{1'}$), 3.12 - 2.88 (m, 4H, H$_3$, CH$_2$, ^{i}Bu, 4-CH$_2$), 2.79 -2.51 (m, 2H, H$_{3'}$), 2.15 - 1.96 (m, 2H, H$_{1'}$, CH$_2$, ^{i}Bu), 1.27 (m, 1H, CH, ^{i}Bu), 0.55 (d, *J* = 6.6 Hz, 3H, CH$_3$, ^{i}Bu), 0.43 (d, *J* = 6.6 Hz, 3H, CH$_3$, ^{i}Bu). ^{13}C-NMR (75 MHz, CDCl$_3$): δ 171.5 (4-CONH), 167.8 (C$_2$), 141.4, 137.8, 136.7, 136.3, 130.6, 130.3, 129.4, 128.7, 128.6, 128.0, 127.3, 127.2, 127.0, 94.7 (Ar), 64.3 (C$_4$), 55.2 (C$_{2'}$), 52.4 (NHCH$_2$), 49.8 (CH$_2$, ^{i}Bu), 49.6 (C$_3$), 47.0 (C$_{1'}$), 41.0 (4-CH$_2$), 38.7 (C$_{3'}$), 28.0 (CH, ^{i}Bu), 20.2, 20.0 (CH$_3$, ^{i}Bu). MS(ES)$^+$: 610.33 [M+H]$^+$. Exact mass calculated for $C_{31}H_{36}IN_3O_2$: 609.18522, found 609.18554.

4*R*,*S*-Benzyl-4-[*N*-(*iso*-butyl)carbamoyl]-1-[(2'*S*-(3''-methoxybenzyl)amino-3'- phenyl)prop-1'-yl]-2-oxoazetidine (25a,b)

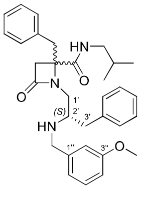

Syrup. Yield: 46% (From **12a,b** and 3-methoxybenzaldehyde). Eluent: 0.9% of MeOH in DCM. HPLC: t$_R$ = 6.39 (m, **25a**) y 6.63 (M, **25 b**) min (gradient from 15% to 95% of A in 10 min). Ratio of diastereoisomers M:m, 2.5:1. ^{1}H-NMR (300 MHz, CDCl$_3$, major diastereoisomer **25b**, from the mixture): δ 10.04 (s, 1H, 4-CONH), 7.40 - 6.36 (m, 15H, Ar, 2'-NH), 3.93 (d, *J* = 14.4 Hz, 1H, 4-CH$_2$), 3.67 (s, 3H, OCH$_3$), 3.64 (dd, *J* = 14.9, 9.0 Hz, 1H, H$_{1'}$), 3.34 (d, *J* = 12.9 Hz, 1H, NHC*H*$_2$), 3.33 (d, *J* = 13.1 Hz, 1H, NHC*H*$_2$), 3.19 - 2.96 (m, 5H, CH$_2$, ^{i}Bu, H$_{1'}$, H$_3$, 4-CH$_2$), 2.77 -2.61 (m, 2H, H$_{2'}$, H$_{3'}$), 2.61 -2.50 (m, 2H, CH$_2$, ^{i}Bu, H$_{3'}$), 1.56 (m, 1H, CH, ^{i}Bu), 0.74 (d, *J* = 6.7 Hz, 3H, CH$_3$, ^{i}Bu), 0.72 (d, *J* = 6.6 Hz, 3H, CH$_3$, ^{i}Bu). ^{13}C-NMR (75 MHz, CDCl$_3$): δ 172.6 (4-CONH), 169.4 (C$_2$), 159.9, 140.0, 137.8, 136.6, 130.2, 129.7, 129.5, 129.0, 129.0, 128.9, 127.4, 127.0, 120.3, 113.6 (Ar), 65.3 (C$_4$),

56.4 ($C_{2'}$), 55.3 (OCH_3), 50.2 (C_3), 48.1 ($NHCH_2$), 47.0 (CH_2, ^{i}Bu), 43.1 ($C_{1'}$), 40.4 (4-CH_2), 38.3 ($C_{3'}$), 28.3 (CH, ^{i}Bu), 20.3, 20.2 (CH_3, ^{i}Bu). ^{1}H-NMR (300 MHz, $CDCl_3$, minor diastereoisomer **25a**, from the mixture): δ 9.57 (s, 1H, 4-CONH), 7.40 - 6.41 (m, 15H, Ar, 2'-NH), 3.87 (d, J = 14.0 Hz, 4-CH_2), 3.79 (m, 1H, $H_{2'}$), 3.67 (s, 3H, OCH_3), 3.62 (d, J = 11.6 Hz, 1H, NHCH_2), 3.56 (d, J = 11.8 Hz, 1H, NHCH_2), 3.24 (dd, J = 14.2, 3.0 Hz, 1H, $H_{1'}$), 3.19 - 2.96 (m, 3H, H_3, CH_2, ^{i}Bu), 2.92 (d, J = 14.1 Hz, 1H, 4-CH_2), 2.68 (m, 1H, $H_{3'}$), 2.55 (m, 1H, $H_{3'}$), 2.06 – 1.90 (m, 2H, $H_{1'}$, CH_2, ^{i}Bu), 1.25 (m, 1H, CH, ^{i}Bu), 0.50 (d, J = 6.6 Hz, 3H, CH_3, ^{i}Bu), 0.37 (d, J = 6.6 Hz, 3H, CH_3, ^{i}Bu). ^{13}C-NMR (75 MHz, $CDCl_3$): δ 171.5 (4-CONH), 167.8 (C_2), 160.0, 140.6, 136.4, 136.1, 130.3, 129.9, 129.3, 128.7, 128.6, 127.3, 127.1, 121.0, 114.6, 112.9 (Ar), 64.3 (C_4), 55.3 ($C_{2'}$), 55.1 (OCH_3), 53.1 ($NHCH_2$), 49.9 (CH_2, ^{i}Bu), 49.6 (C_3), 46.5 ($C_{1'}$), 41.1 (4-CH_2), 38.5 ($C_{3'}$), 27.8 (CH, ^{i}Bu), 20.1, 19.9 (CH_3, ^{i}Bu). MS(ES)$^+$: 514.33 [M+H]$^+$. Exact mass calculated for $C_{32}H_{39}N_3O_3$: 513.29914, found 513.29984.

4*R*,*S*-Benzyl-4-[*N*-(*iso*-butyl)carbamoyl]-1-[(2'*S*-(3''-benzyloxibenzyl)amino-3'- phenyl)prop-1'-yl]-2-oxoazetidine (26a,b)

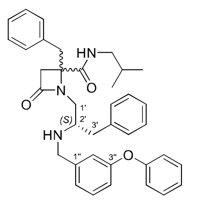

Sirup. Yield: 24% (From **12a,b** and 3-benzyloxybenzaldehyde). Eluent: 9% to 33% of EtOAc in DCM. HPLC: t_R = 7.18 (m, **26a**) y 7.34 (M, **26 b**) min (gradient from 15% to 95% of A in 10 min). Ratio of diastereoisomers M:m, 5.7:1. ^{1}H-NMR (300 MHz, $CDCl_3$, major diastereoisomer): δ 9.98 (s, 1H, 4-CONH), 7.53 - 6.65 (m, 20H, Ar, 2'-NH), 3.93 (d, J = 14.3 Hz, 1H, 4-CH_2), 3.60 (dd, J = 15.0, 6.0 Hz, 1H, $H_{1'}$), 3.32 (d, J = 13.1 Hz, 1H, NHCH_2), 3.26 (d, J = 13.1 Hz, 1H, NHCH_2), 3.07 - 2.88 (m, 5H, CH_2, ^{i}Bu, $H_{1'}$, H_3, 4-CH_2), 2.76 -2.51 (m, 2H, $H_{2'}$, $H_{3'}$), 2.61 - 2.50 (m, 2H, CH_2, ^{i}Bu, $H_{3'}$), 1.58 (m, 1H, CH, ^{i}Bu), 0.75 (t, J = 6.7 Hz, 6H, CH_3, ^{i}Bu). ^{13}C-NMR (75 MHz, $CDCl_3$, major diastereoisomer): δ 172.5 (4-CONH), 169.4 (C_2), 157.6, 157.0, 140.4, 137.7, 136.5, 130.2, 130.0, 129.9, 128.9, 128.9, 128.8, 127.4, 127.0, 123.6, 122.6, 119.1, 118.3, 117.8 (Ar), 65.2 (C_4), 56.3 ($C_{2'}$), 50.2 (C_3), 47.7 ($NHCH_2$), 47.0 (CH_2, ^{i}Bu), 43.0 ($C_{1'}$), 40.5 (4-CH_2), 38.3 ($C_{3'}$), 28.3 (CH, ^{i}Bu), 20.4, 20.3 (CH_3, ^{i}Bu). MS(ES)$^+$: 576.26 [M+H]$^+$. Exact mass calculated for $C_{37}H_{41}N_3O_3$: 575.31479, found 575.31707.

4*R*,*S*-Benzyl-4-[*N*-(*iso*-butyl)carbamoyl]-1-[(2'*S*-(3''-phenoxybenzyl)amino-3'- phenyl)prop-1'-yl]-2-oxoazetidine (27a,b)

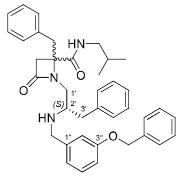

Syrup. Yield: 22% (From **12a,b** and 3-phenoxybenzaldehyde. Eluent: 9% to 20% of EtOAc in DCM. HPLC: t_R = 7.26 (m, **27a**) y 7.47 (M, **27b**) min (gradient from 15% to 95% of A in 10 min). Ratio of diastereoisomers M:m, 1.5:1. ^{1}H-NMR (300 MHz, $CDCl_3$, major diastereoisomer **27b**, from the mixture): δ 10.06 (t, J = 5.9 Hz, 1H, 4-CONH), 7.49 - 6.34 (m, 20H, Ar, 2'-NH), 4.95 (s, 2H, OCH_2), 3.93 (d, J = 14.3 Hz, 1H, 4-CH_2), 3.61 (dd, J = 14.9, 5.7 Hz, 1H, $H_{1'}$), 3.37 (d, J = 12.9 Hz, 1H, NHCH_2), 3.30 (d, J = 13.1 Hz, 1H, NHCH_2), 3.07 - 2.87 (m, 5H, CH_2, ^{i}Bu, $H_{1'}$, H_3, 4-CH_2), 2.75 - 2.49 (m, 4H, $H_{2'}$, CH_2, ^{i}Bu, $H_{3'}$), 1.58 (m, 1H, CH, ^{i}Bu), 0.75 (d, J = 6.7 Hz, 3H, CH_3, ^{i}Bu), 0.73 (d, J = 6.7 Hz, 3H, CH_3, ^{i}Bu). ^{13}C-NMR (75 MHz, $CDCl_3$): δ 172.6 (4-CONH), 169.7 (C_2), 159.1, 140.1, 137.8, 137.0,

136.6, 130.2, 129.8, 129.1, 129.0, 128.9, 128.7, 128.6, 127.5, 127.4, 127.0, 120.6, 114.6, 113.7 (Ar), 70.0 (OCH_2), 65.3 (C_4), 56.3 ($C_{2'}$), 50.1 (C_3), 48.1 ($NHCH_2$), 47.0 (CH_2, ^{i}Bu), 43.2 ($C_{1'}$), 40.4 (4-CH_2), 38.3 ($C_{3'}$), 28.3 (CH, ^{i}Bu), 20.4, 20.3 (CH_3, ^{i}Bu). ^{1}H-NMR (300 MHz, $CDCl_3$, major diastereoisomer **27a**, from the mixture): δ 9.58 (t, J = 5.7 Hz, 1H, 4-CONH), 7.49 - 6.82 (m, 20H, Ar, 2'-NH), 5.01 (s, 2H, OCH_2), 3.87 (d, J = 14.0 Hz, 4-CH_2), 3.78 (m, 1H, $H_{2'}$), 3.70 (d, J = 11.9 Hz, 1H, NHCH_2), 3.57 (d, J = 11.9 Hz, 1H, NHCH_2), 3.17 (dd, J = 13.4, 6.9 Hz, 1H, $H_{1'}$), 3.07 - 2.87 (m, 5H, CH_2, ^{i}Bu, $H_{1'}$, H_3, 4-CH_2), 2.75 - 2.49 (m, 2H, $H_{3'}$), 2.09 - 1.91 (m, 2H, $H_{1'}$, CH_2, ^{i}Bu), 1.26 (m, 1H, CH, ^{i}Bu), 0.50 (d, J = 6.7 Hz, 3H, CH_3, ^{i}Bu), 0.38 (d, J = 6.6 Hz, 3H, CH_3, ^{i}Bu). ^{13}C-NMR (75 MHz, $CDCl_3$): δ 171.5 (4-CONH), 167.5 (C_2), 159.2, 140.7, 136.9, 136.4, 136.2, 130.3, 130.0, 129.5, 129.0, 128.8, 128.8, 128.7, 128.2, 127.6, 127.6, 127.1, 121.3, 115.5 (Ar), 70.1 (OCH_2), 64.3 (C_4), 55.1 ($C_{2'}$), 53.1 ($NHCH_2$), 49.9 (CH_2, ^{i}Bu), 49.6 (C_3), 46.5 ($C_{1'}$), 41.1 (4-CH_2), 38.6 ($C_{3'}$), 27.9 (CH, ^{i}Bu), 20.1, 20.0 (CH_3, ^{i}Bu). MS(ES)$^+$: 590.50 $[M+H]^+$. Exact mass calculated for $C_{38}H_{43}N_3O_3$: 589.33044, found 589.32992.

4*R*,*S*-Benzyl-4-[*N-(iso*-butyl)carbamoyl]-1-[(2'*S*-(3''-cyanobenzyl)amino-3'- phenyl)prop -1'-yl]-2-oxoazetidine (28a,b)

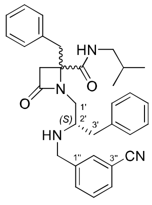

Syrup. Yield: 24% (From **12a,b** and 3-cyanobenzaldehyde). Eluent: 9% to 11% of EtOAc in DCM. HPLC: t_R = 6.32 (m, **28a**) y 6.57 (M, 2**8b**) min (gradient from 15% to 95% of A in 10 min). Ratio of diastereoisomers M:m, 1.1:1. ^{1}H-NMR (300 MHz, $CDCl_3$, major diastereoisomer **28b**, from the mixture): δ 9.53 (t, J = 5.8 Hz, 1H, 4-CONH), 7.62 - 6.83 (m, 15H, Ar, 2'-NH), 3.94 (d, J = 14.3 Hz, 1H, 4-CH_2), 3.61 (dd, J = 15.1, 6.3 Hz, 1H, $H_{1'}$), 3.46 (d, J = 13.3 Hz, 1H, NHCH_2), 3.39 (d, J = 13.3 Hz, 1H, NHCH_2), 3.22 - 2.90 (m, 5H, CH_2, ^{i}Bu, $H_{1'}$, H_3, 4-CH_2), 2.76 - 2.56 (m, 4H, $H_{2'}$, CH_2, ^{i}Bu, $H_{3'}$), 1.58 (m, 1H, CH, ^{i}Bu), 0.76 (d, J = 6.7 Hz, 3H, CH_3, ^{i}Bu), 0.75 (d, J = 6.7 Hz, 3H, CH_3, ^{i}Bu). ^{13}C-NMR (75 MHz, $CDCl_3$): δ 172.4 (4-CONH), 169.3 (C_2), 140.2, 137.5, 136.0, 132.6, 131.4, 131.2, 130.2, 129.5, 129.2, 128.9, 128.9, 127.5, 127.3 (Ar), 118.5 (CN), 113.0 (Ar), 65.2 (C_4), 56.1 ($C_{2'}$), 50.2 (C_3), 47.1 ($NHCH_2$), 46.9 (CH_2, ^{i}Bu), 43.0 ($C_{1'}$), 40.4 (4-CH_2), 38.2 ($C_{3'}$), 28.4 (CH, ^{i}Bu), 20.4, 20.3 (CH_3, ^{i}Bu). ^{1}H-NMR (300 MHz, $CDCl_3$, minor diastereoisomer **28a**, from the mixture): δ 9.17 (t, J = 5.8 Hz, 1H, 4-CONH), 7.62 - 6.83 (m, 15H, Ar, 2'-NH), 3.84 (d, J = 14.0 Hz, 4-CH_2), 3.74 (m, 1H, $H_{2'}$), 3.68 (d, J = 11.9 Hz, 1H, NHCH_2), 3.57 (d, J = 11.9 Hz, 1H, NHCH_2), 3.28 (dd, J = 14.3, 3.1 Hz, 1H, H_1), 3.22 - 2.90 (m, 4H, H_3, CH_2, ^{i}Bu, 4-CH_2), 2.62 (m, 1H, $H_{3'}$), 2.46 (dd, J = 13.9, 6.1 Hz, 1H, $H_{3'}$), 2.23 - 2.03 (m, 2H, $H_{1'}$, CH_2, ^{i}Bu), 1.33 (m, 1H, CH, ^{i}Bu), 0.57 (d, J = 6.5 Hz, 3H, CH_3, ^{i}Bu), 0.47 (d, J = 6.5 Hz, 3H, CH_3, ^{i}Bu). ^{13}C-NMR (75 MHz, $CDCl_3$): δ 171.5 (4-CONH), 167.8 (C_2), 140.6, 136.6, 136.2, 133.4, 132.1, 131.3, 130.3, 129.7, 129.3, 129.0, 128.7, 127.4, 127.3 (Ar), 118.51 (CN), 112.81 (Ar), 64.4 (C_4), 55.0 ($C_{2'}$), 52.2 ($NHCH_2$), 49.8 (CH_2, ^{i}Bu), 49.4 (C_3), 46.8 ($C_{1'}$), 41.0 (4-CH_2), 38.9 ($C_{3'}$), 28.1 (CH, ^{i}Bu), 20.2, 20.0 (CH_3, ^{i}Bu). MS(ES)$^+$: 509.36 $[M+H]^+$. Exact mass calculated for $C_{32}H_{36}N_4O_2$: 508.28383, found 508.2845.

4*R*,*S*-Benzyl-4-[*N-(iso*-butyl)carbamoyl]-1-[(2'*S*-(3''-nitrobenzyl)amino-3'- phenyl)prop -1'-yl]-2-oxoazetidine (29a,b)

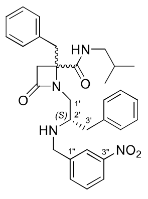

Syrup. Yield: 16% (From **12a,b** and 3-nitrobenzaldehyde). Eluent: 9% to 50% of EtOAc in DCM. HPLC: t_R = 6.47 (M, **29a**) y 6.73 (m, **29b**) min (gradient from 15% to 95% of A in 10 min). Ratio of diastereoisomers M:m, 1.2:1. ^{1}H-NMR (300 MHz, $CDCl_3$, major diastereoisomer **29a**, from the mixture): δ 9.19 (t, J = 5.2 Hz, 1H, 4-CONH), 8.16 - 6.84 (m, 15H, Ar, 2'-NH), 3.83 (d, J = 14.0 Hz, 4-CH_2), 3.76 (s, 2H, NHCH_2), 3.73 (m, 1H, $H_{2'}$), 3.29 (dd, J = 14.0, 3.1 Hz, 1H, $H_{1'}$), 3.20 - 2.90 (m, 4H, H_3, CH_2, ^{i}Bu, 4-CH_2), 2.65 (m, 1H, $H_{3'}$), 2.48 (dd, J = 13.9, 6.2 Hz, 1H, $H_{3'}$), 2.27 - 2.02 (m, 2H, $H_{1'}$, CH_2, ^{i}Bu), 1.38 (m, 1H, CH, ^{i}Bu), 0.58 (d, J = 6.6 Hz, 3H, CH_3, ^{i}Bu), 0.48 (d, J = 6.6 Hz, 3H, CH_3, ^{i}Bu). ^{13}C-NMR (75 MHz, $CDCl_3$): δ 171.5 (4-CONH), 167.9 (C_2), 148.6, 140.7, 136.5, 135.9, 135.1, 130.3, 129.8, 129.3, 129.1, 128.7, 127.4, 127.3, 123.4, 122.7 (Ar), 64.4 (C_4), 54.9 ($C_{2'}$), 51.9 ($NHCH_2$), 49.8 (CH_2, ^{i}Bu), 49.4 (C_3), 46.8 ($C_{1'}$), 41.0 (4-CH_2), 38.8 ($C_{3'}$), 28.2 (CH, ^{i}Bu), 20.2, 20.1 (CH_3, ^{i}Bu). ^{1}H-NMR (300 MHz, $CDCl_3$, minor diastereoisomer **29b**, from the mixture): δ 9.46 (s, 1H, 4-CONH), 8.16 - 6.84 (m, 15H, Ar, 2'-NH), 3.94 (d, J = 14.3 Hz, 1H, 4-CH_2), 3.64 (dd, J = 15.1, 7.0 Hz, 1H, $H_{1'}$), 3.54 (d, J = 13.5 Hz, 1H, NHCH_2), 3.48 (d, J = 13.7 Hz, 1H, NHCH_2), 3.20 - 2.90 (m, 5H, CH_2, ^{i}Bu, $H_{1'}$, H_3, 4-CH_2), 2.79 - 2.57 (m, 4H, $H_{2'}$, CH_2, ^{i}Bu, $H_{3'}$), 1.59 (m, 1H, CH, ^{i}Bu), 0.74 (d, J = 6.6 Hz, 6H, CH_3, ^{i}Bu). ^{13}C-NMR (75 MHz, $CDCl_3$): δ 172.5 (4-CONH), 169.3 (C_2), 148.5, 141.0, 137.5, 136.1, 134.2, 130.1, 129.7, 129.1, 129.0, 128.9, 127.6, 127.2, 122.9, 122.6 (Ar), 65.2 (C_4), 56.1 ($C_{2'}$), 50.2 (C_3), 49.8 (CH_2, ^{i}Bu), 47.1 ($NHCH_2$), 43.1 ($C_{1'}$), 40.4 (4-CH_2), 38.2 ($C_{3'}$), 28.4 (CH, ^{i}Bu), 20.33, 20.27 (CH_3, ^{i}Bu). MS(ES)$^+$: 529.26 $[M+H]^+$. Exact mass calculated for $C_{31}H_{36}N_4O_4$: 528.27366, found 528.2737.

4*R*,*S*-Benyl-4-[*N*-(*iso*-butyl)carbamoyl]-1-[(2'*S*-(4''-biphenyl)methylamino-3'- phenyl)prop -1'-yl]-2-oxoazetidine (30a,b)

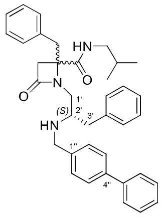

Syrup. Yield: 70% (From **12a,b** 4-biphenyl-carboxaldehyde) Eluent: 9% to 50% of EtOAc in DCM. HPLC: t_R = 7.19 (m, **30a**) y 7.38 (M, **30b**) min (gradient from 15% to 95% of A in 10 min). Ratio of diastereoisomers M:m, 1.9:1. ^{1}H-NMR (300 MHz, $CDCl_3$, major diastereoisomer **30b**, from the mixture): δ 10.06 (t, J = 5.9 Hz, 1H, 4-CONH), 7.59 - 6.86 (m, 20H, Ar, 2'-NH), 3.94 (d, J = 14.4 Hz, 1H, 4-CH_2), 3.42 (s, 2H, NHCH_2), 3.21 - 2.88 (m, 5H, CH_2, ^{i}Bu, $H_{1'}$, H_3, 4-CH_2), 2.78 - 2.51 (m, 4H, $H_{2'}$, CH_2, ^{i}Bu, $H_{3'}$), 1.55 (m, 1H, CH, ^{i}Bu), 0.73 (d, J = 6.8 Hz, 3H, CH_3, ^{i}Bu), 0.71 (d, J = 6.6 Hz, 3H, CH_3, ^{i}Bu). ^{13}C-NMR (75 MHz, $CDCl_3$): δ 172.5 (4-CONH), 169.4 (C_2), 140.7, 137.8, 136.5, 136.1, 130.2, 129.0, 128.9, 1289, 128.8, 128.4, 127.5, 127.4, 127.1, 127.1 (Ar), 65.2 (C_4), 56.4 ($C_{2'}$), 50.1 (C_3), 47.6 ($NHCH_2$), 47.0 (CH_2, ^{i}Bu), 43.2 ($C_{1'}$), 40.3 (4-CH_2), 38.3 ($C_{3'}$), 28.2 (CH, ^{i}Bu), 20.3, 20.2 (CH_3, ^{i}Bu). ^{1}H-NMR (300 MHz, $CDCl_3$, minor diastereoisomer **30a**, from the mixture): 9.59 (t, J = 6.1 Hz, 1H, 4-CONH), 7.59 - 6.90 (m, 20H, Ar, 2'-NH), 3.88 (d, J = 14.0 Hz, 4-CH_2), 3.82 (m, 1H, $H_{2'}$), 3.77 (d, J = 11.9 Hz, 1H, NHCH_2), 3.65 (m, 2H, $H_{1'}$, NHCH_2), 3.26 (dd, J = 14.1, 2.9 Hz, 1H, $H_{1'}$), 3.21 - 2.88 (m, 4H, H_3, CH_2, ^{i}Bu, 4-CH_2), 2.78 - 2.51 (m, 2H, $H_{3'}$), 2.10 - 1.89 (m, 2H, $H_{1'}$, CH_2, ^{i}Bu),

1.25 (m, 1H, CH, ^{i}Bu), 0.46 (d, *J* = 6.6 Hz, 3H, CH_3, ^{i}Bu), 0.34 (d, *J* = 6.6 Hz, 3H, CH_3, ^{i}Bu). ^{13}C-NMR (75 MHz, $CDCl_3$): δ 171.4 (4-CONH), 167.8 (C_2), 140.7, 140.4, 138.1, 137.5, 136.4, 130.3, 129.5, 129.2, 128.9, 128.6, 127.6, 127.4, 127.2, 126.9 (Ar), 64.3 (C_4), 55.1 ($C_{2'}$), 52.8 ($NHCH_2$), 49.9 (CH_2, ^{i}Bu), 49.6 (C_3), 46.5 ($C_{1'}$), 41.3 (4-CH_2), 38.6 ($C_{3'}$), 27.8 (CH, ^{i}Bu), 20.1, 19.9 (CH_3, ^{i}Bu). MS(ES)$^+$: 560.49 $[M+H]^+$. Exact mass calculated for $C_{37}H_{41}N_3O_2$: 559.31988, found 559.32217.

4*R*,*S*-Benzyl-4-[*N-(iso*-butyl)carbamoyl]-1-[(2'*S*-(4''-fluorobenzyl)amino-3'- phenyl)prop-1'-yl]-2-oxoazetidine (31a,b)

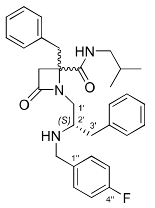

Syrup. Yield: 10% (From **12a,b** and 4-fluorobenzaldehyde). Eluent: 9% to 33% of EtOAc in DCM. HPLC: t_R = 6.45 (M, **31a**) y 6.70 (m,**31b**) min (gradient from 15% to 95% of A in 10 min). Ratio of diastereoisomers M:m, 1.1:1. ^{1}H-NMR (300 MHz, $CDCl_3$, major diastereoisomer **31a**, from the mixture): δ 9.50 (s, 1H, 4-CONH), 7.38 - 6.75 (m, 15H, Ar, 2'-NH), 3.87 (d, *J* = 13.9 Hz, 4-CH_2), 3.78 (m, 1H, $H_{2'}$), 3.67 (d, *J* = 12.1 Hz, 1H, NHC*H*$_2$), 3.58 (d, *J* = 12.2 Hz, 1H, NHC*H*$_2$), 3.25 (dd, *J* = 14.0, 3.0 Hz, 1H, $H_{1'}$), 3.18 - 2.85 (m, 4H, H_3, CH_2, ^{i}Bu, 4-CH_2), 2.62 (m, 1H, $H_{3'}$), 2.51 (dd, *J* = 13.7, 5.9 Hz, 1H, $H_{3'}$), 2.11 - 1.92(m, 2H, $H_{1'}$, CH_2, ^{i}Bu), 1.24 (m, 1H, CH, ^{i}Bu), 0.52 (d, *J* = 6.7 Hz, 3H, CH_3, ^{i}Bu), 0.40 (d, *J* = 6.6 Hz, 3H, CH_3, ^{i}Bu). ^{13}C-NMR (75 MHz, $CDCl_3$): δ 171.5 (4-CONH), 167.8 (C_2), 162.3 (d, *J* = 246.3 Hz, $C_{4''}$), 137.8, 136.1, 135.0 (d, *J* = 2.8 Hz, $C_{1''}$), 130.3, 130.2, 129.4, 129.0, 128.9, 128.6, 127.4 (d, *J* = 10.4 Hz, $C_{2''}$, $C_{6''}$), 115.7 (d, *J* = 21.4 Hz, $C_{3''}$, $C_{5''}$) (Ar), 64.4 (C_4), 55.0 ($C_{2'}$), 52.3 ($NHCH_2$), 49.9 (CH_2, ^{i}Bu), 49.6 (C_3), 46.6 ($C_{1'}$), 41.0 (4-CH_2), 38.1 ($C_{3'}$), 27.9 (CH, ^{i}Bu), 20.1, 20.0 (CH_3, ^{i}Bu). ^{1}H-NMR (300 MHz, $CDCl_3$, minor diastereoisomer **31b**, from the mixture): δ 9.90 (s, 1H, 4-CONH), 7.38 - 6.75 (m, 15H, Ar, 2'-NH), 3.94 (d, *J* = 14.3 Hz, 1H, 4-CH_2), 3.64 (dd, *J* = 14.7, 6.8 Hz, 1H, $H_{1'}$), 3.36 (s, 2H, NHC*H*$_2$), 3.18 - 2.85 (m, 5H, CH_2, ^{i}Bu, $H_{1'}$, H_3, 4-CH_2), 2.75 - 2.56 (m, 4H, $H_{2'}$, CH_2, ^{i}Bu, $H_{3'}$), 1.54 (m, 1H, CH, ^{i}Bu), 0.73 (d, *J* = 6.7 Hz, 3H, CH_3, ^{i}Bu), 0.72 (d, *J* = 6.7 Hz, 3H, CH_3, ^{i}Bu).^{13}C-NMR (75 MHz, $CDCl_3$): δ 172.6 (4-CONH), 169.4 (C_2), 162.2 (d, *J* = 245.9 Hz, $C_{4''}$), 136.6, 136.3, 134.3 (d, *J* = 2.8 Hz, $C_{1''}$), 130.5, 130.4, 129.7, 129.6, 129.0, 128.9, 127.1 (d, *J* = 9.2 Hz, $C_{2''}$, $C_{6''}$), 115.5 (d, *J* = 21.3 Hz, $C_{3''}$, $C_{5''}$) (Ar), 65.2 (C_4), 56.2 ($C_{2'}$), 50.2 (C_3), 47.05 ($NHCH_2$), 47.02 (CH_2, ^{i}Bu), 43.1 ($C_{1'}$), 40.3 (4-CH_2), 38.1 ($C_{3'}$), 28.3 (CH, ^{i}Bu), 20.34, 20.26 (CH_3, ^{i}Bu). MS(ES)$^+$: 502.32 $[M+H]^+$. Exact mass calculated for $C_{31}H_{36}FN_3O_2$: 501.27916, found 501.28014.

4*R*,*S*-Benzyl-4-[*N-(iso*-butyl)carbamoyl]-1-[(2'*S*-naphtylmethylamino-3'- phenyl)prop-1'-yl]-2-oxoazetidine (32,ab)

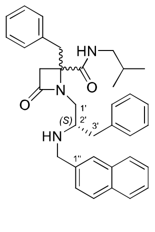

Syrup. Yield: 80% (From **12a,b** and 2-naphtaldehyde). Eluent: 9% to 50% of EtOAc in DCM. HPLC: t_R = 6.83 (m, **32a**) y 7.07 (M, **32b**) min (gradient from 15% to 95% of A in 10 min). Ratio of diastereoisomers M:m, 1.1:1. ^{1}H-NMR (300 MHz, $CDCl_3$, major diastereoisomer **32b**, from the mixture): δ 10.07 (t, *J* = 5.9 Hz, 1H, 4-CONH), 7.84 - 6.83 (m, 18H, Ar, 2'-NH), 3.96 (d, *J* = 14.3 Hz,

1H, 4-CH_2), 3.63 (dd, J = 14.9, 5.7 Hz, 1H, $H_{1'}$), 3.55 (d, J = 13.1 Hz, 1H, NHCH_2), 3.47 (d, J = 13.1 Hz, 1H, NHCH_2), 3.19 - 2.87 (m, 5H, CH_2, ^{i}Bu, $H_{1'}$, H_3, 4-CH_2), 2.80 - 2.51 (m, 4H, $H_{2'}$, CH_2, ^{i}Bu, $H_{3'}$), 1.53 (m, 1H, CH, ^{i}Bu), 0.70 (d, J = 6.7 Hz, 3H, CH_3, ^{i}Bu), 0.66 (d, J = 6.6 Hz, 3H, CH_3, ^{i}Bu). ^{13}C-NMR (75 MHz, $CDCl_3$): δ 172.5 (4-CONH), 169.4 (C_2), 137.8, 136.3, 136.1, 133.3, 132.8, 130.2, 129.4, 128.9, 128.8, 128.6, 127.7, 127.6, 127.4, 127.0, 126.7, 126.4, 126.1, 126.0 (Ar), 65.2 (C_4), 56.3 ($C_{2'}$), 50.1 (C_3), 48.1 (NHCH_2), 46.9 (CH_2, ^{i}Bu), 43.1 ($C_{1'}$), 40.5 M (4-CH_2), 38.4 ($C_{3'}$), 28.2 (CH, ^{i}Bu), 20.21, 20.15 (CH_3, ^{i}Bu). ^{1}H-NMR (300 MHz, $CDCl_3$, minor diastereoisomer **32a**, from the mixture): δ 9.53 (t, J = 5.6 Hz, 1H, 4-CONH), 7.84 - 6.83 (m, 18H, Ar, 2'-NH), 3.86 (m, 3H, 4-CH_2, $H_{2'}$, NHCH_2), 3.78 (d, J = 12.0 Hz, 1H, NHCH_2), 3.27 (dd, J = 14.0, 3.0 Hz, 1H, $H_{1'}$), 3.19 - 2.87 (m, 4H, H_3, CH_2, ^{i}Bu, 4-CH_2), 2.80 - 2.51 (m, 6H, $H_{3'}$), 1.96 – 1.87 (m, 2H, $H_{1'}$, CH_2, ^{i}Bu), 1.08 (m, 1H, CH, ^{i}Bu), 0.32 (d, J = 6.6 Hz, 3H, CH_3, ^{i}Bu), 0.18 (d, J = 6.6 Hz, 3H, CH_3, ^{i}Bu). ^{13}C-NMR (75 MHz, $CDCl_3$): δ 171.4 (4-CONH), 167.7 (C_2), 136.6, 136.4, 135.7, 133.5, 132.7, 130.2, 129.0, 128.8, 128.6, 128.4, 127.7, 127.4, 127.2, 126.9, 126.5, 126.3, 126.0 (Ar), 64.3 (C_4), 55.2 ($C_{2'}$), 53.2 (NHCH_2),49.8 (CH_2, ^{i}Bu), 49.5 (C_3), 46.4 ($C_{1'}$), 41.0 (4-CH_2), 38.6 ($C_{3'}$), 27.7 (CH, ^{i}Bu), 19.75, 19.62 (CH_3, ^{i}Bu). MS(ES)$^+$: 534.46 $[M+H]^+$. Exact mass calculated for $C_{35}H_{39}N_3O_2$: 533.30423, found 533.30655.

4*S*-Benzyl-4-carboxy-3*S*-methyl-1-[(2'*S*-dibenzylamino-3'-phenyl)prop-1'-yl]-2-oxoazetidine (33)

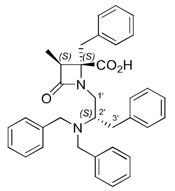

White solid, M.p.: 175.9 ºC (EtOAc:hexane). Yield: 72% (From **1**). Eluent: 2% to 9% of MeOH in DCM. HPLC: t_R = 6.84 min (gradient from 10% to 95% of A in 10 min). ^{1}H-NMR (400 MHz, $CDCl_3$): δ 7.58 (s ancho, 4H, Ar), 7.44 (s ancho, 6H, Ar), 7.22 - 7.09 (m, 3H, Ar), 6.96 (m, 2H, Ar), 6.82 (m, 1H, Ar), 6.63 (m, 2H, Ar), 6.38 (m, 2H, Ar), 4.43 (s ancho, 1H, COOH), 4.06 (d, J = 14.3 Hz, 2H, NCH_2), 3.86 (d, J = 13.1 Hz, 1H, 4-CH_2), 3.82 (d, J = 12.8 Hz, 1H, 4-CH_2), 3.60 (m, 2H, $H_{1'}$, $H_{2'}$), 3.16 (q, J = 7.5 Hz, 1H, H_3), 3.13 (m, 1H, $H_{3'}$), 2.70 (d, J = 14.3 Hz, NCH_2), 2.69 (m, 1H, $H_{1'}$), 2.46 (dd, J = 12.8, 10.9 Hz, 1H, $H_{3'}$), 1.44 (d, J = 7.5 Hz, 3H, 3-CH_3). ^{13}C-NMR (75 MHz, $CDCl_3$): δ 174.1 (COOH), 173.3 (C_2), 136.8, 134.1, 131.1, 130.5, 129.5, 129.4, 129.1, 128.9, 128.8, 128.7, 127.2, 126.7 (Ar), 72.6 (C_4), 56.6 ($C_{2'}$), 55.0 (NCH_2), 42.0 ($C_{1'}$), 40.4 ($C_{3'}$, 4-CH_2), 31.5 ($C_{3'}$), 10.5 (3-CH_3). MS(ES)$^+$: 533.11 $[M+H]^+$.

Table 3S. Yield and **a**:**b** ratio diastereoisomers of *N*-monobenzylamines **34-45**.

Compound	R^1	R^2	Ratio of diastereoisomers (a:b)	Yields	Yield of 2.17a
**11ab**	OMe	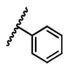	1:3.6	40%	6%
**13ab**	OMe	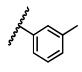	1:3.6	30%	4%
**14ab**	OMe	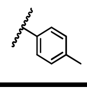	1:5.6	30%	4%

**15ab**	OMe	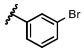	1:2	40%	12%
**16ab**	OMe	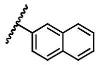	1:4	45%	9%
**17ab**	OMe	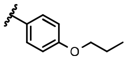	1:9.5	36%	9%
**18ab**	NH^{i}Bu	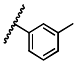	1:1.5	28%	-
**19ab**	NH^{i}Bu	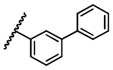	1:1.1	80%	-
**20ab**	NH^{i}Bu	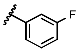	1:2.6	32%	-
**21ab**	NH^{i}Bu	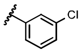	1:2.4	27%	-
**22ab**	NH^{i}Bu	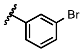	1:2.9	30%	-
**23ab**	NH^{i}Bu	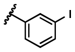	1:2.7	19%	-
**24ab**	NH^{i}Bu	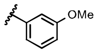	1:2.5	46%	-
**25ab**	NH^{i}Bu	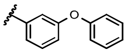	1:5.7	24%	-
**26ab**	NH^{i}Bu	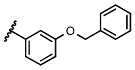	1:1.5	22%	-
**27ab**	NH^{i}Bu	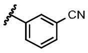	1:1.1	24%	-
**28ab**	NH^{i}Bu	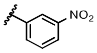	1.2:1	16%	-
**29ab**	NH^{i}Bu	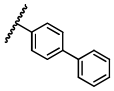	1:1.9	70%	-
**30ab**	NH^{i}Bu	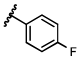	1.1:1	10%	-
**31ab**	NH^{i}Bu	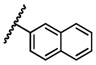	1:1.1	80%	-

Modeling Studies

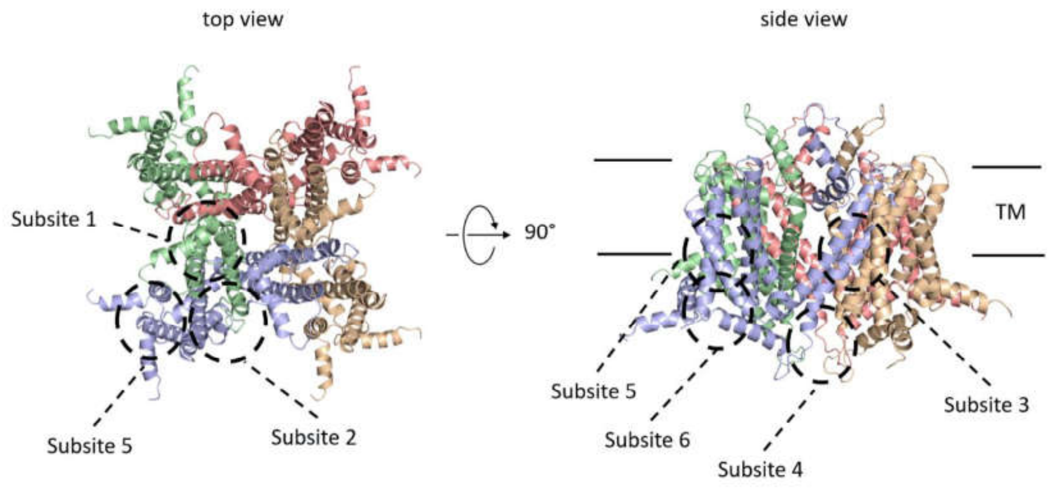

Figure S1. Comparative location of subsites 1-6 within the *r*TRPM8 structure

```
TRPM8_HUMAN
MSFRAARLSMRNRRNDTLDSTRTLYSSASRSTDLSYSESDLVNFIQANFKKRECVFFTKD        60
TRPM8_RAT
MSFEGARLSMRSRRNGTLGSTRTLYSSVSRSTDVSYSESDLVNFIQANFKKRECVFFTRD        60
                 ***..******.***.**.********.*****:************************:*

TRPM8_HUMAN
SKATENVCKCGYAQSQHMEGTQINQSEKWNYKKHTKEFPTDAFGDIQFETLGKKGKYIRL        120
TRPM8_RAT
SKAMESICKCGYAQSQHIEGTQINQNEKWNYKKHTKEFPTDAFGDIQFETLGKKGKYLRL        120
                 *** *.:**********:*******.*******************************:**

TRPM8_HUMAN
SCDTDAEILYELLTQHWHLKTPNLVISVTGGAKNFALKPRMRKIFSRLIYIAQSKGAWIL        180
TRPM8_RAT
SCDTDSETLYELLTQHWHLKTPNLVISVTGGAKNFALKPRMRKIFSRLIYIAQSKGAWIL        180
                 *****:* ****************************************************

TRPM8_HUMAN
TGGTHYGLMKYIGEVVRDNTISRSSEENIVAIGIAAWGMVSNRDTLIRNCDAEGYFLAQY        240
TRPM8_RAT
TGGTHYGLMKYIGEVVRDNTISRNSEENIVAIGIAAWGMVSNRDTLIRNCDDEGHFSAQY        240
                 ***********************.*************************** **:* ***

TRPM8_HUMAN
LMDDFTRDPLYILDNNHTHLLLVDNGCHGHPTVEAKLRNQLEKYISERTIQDSNYGGKIP        300
TRPM8_RAT
IMDDFMRDPLYILDNNHTHLLLVDNGCHGHPTVEAKLRNQLEKYISERTSQDSNYGGKIP        300
                 :**** ******************************************* **********

TRPM8_HUMAN
IVCFAQGGGKETLKAINTSIKNKIPCVVVEGSGQIADVIASLVEVEDALTSSAVKEKLVR        360
TRPM8_RAT
IVCFAQGGGRETLKAINTSVKSKIPCVVVEGSGQIADVIASLVEVEDVLTSSMVKEKLVR        360
                 *********:*********:*.*************************.**** *******

TRPM8_HUMAN
FLPRTVSRLPEEETESWIKWLKEILECSHLLTVIKMEEAGDEIVSNAISYALYKAFSTSE        420
TRPM8_RAT
FLPRTVSRLPEEEIESWIKWLKEILESPHLLTVIKMEEAGDEVVSSAISYALYKAFSTNE        420
                 ************* ************. **************:**.************.*

TRPM8_HUMAN
QDKDNWNGQLKLLLEWNQLDLANDEIFTNDRRWESADLQEVMFTALIKDRPKFVRLFLEN        480
TRPM8_RAT
QDKDNWNGQLKLLLEWNQLDLASDEIFTNDRRWESADLQEVMFTALIKDRPKFVRLFLEN        480
                 **********************.*************************************

TRPM8_HUMAN
GLNLRKFLTHDVLTELFSNHFSTLVYRNLQIAKNSYNDALLTFVWKLVANFRRGFRKEDR        540
TRPM8_RAT
GLNLQKFLTNEVLTELFSTHFSTLVYRNLQIAKNSYNDALLTFVWKLVANFRRSFWKEDR        540
                 ****:****::*******.**********************************.* ****

TRPM8_HUMAN
NGRDEMDIELHDVSPITRHPLQALFIWAILQNKKELSKVIWEQTRGCTLAALGASKLLKT        600
TRPM8_RAT
SSREDLDVELHDASLTTRHPLQALFIWAILQNKKELSKVIWEQTKGCTLAALGASKLLKT        600
                 ..*:::*:****.*  ****************************:***************

TRPM8_HUMAN
LAKVKNDINAAGESEELANEYETRAVELFTECYSSDEDLAEQLLVYSCEAWGGSNCLELA        660
TRPM8_RAT
LAKVKNDINAAGESEELANEYETRAVELFTECYSSDEDLAEQLLVYSCEAWGGSNCLELA        660
                 ************************************************************
```

```
TRPM8_HUMAN
VEATDQHFIAQPGVQNFLSKQWYGEISRDTKNWKIILCLFIIPLVGCGFVSFRKKPVDKH        720
TRPM8_RAT
VEATDQHFIAQPGVQNFLSKQWYGEISRDTKNWKIILCLFIIPLVGCGLVSFRKKPIDKH        720
                 ************************************************:*******:***

                   Pre-S1                S1                          S2
TRPM8_HUMAN
KKLLWYYVAFFTSPFVVFSWNVVFYIAFLLLFAYVLLMDFHSVPHPPELVLYSLVFVLFC        780
TRPM8_RAT
KKLLWYYVAFFTSPFVVFSWNVVFYIAFLLLFAYVLLMDFHSVPHTPELILYALVFVLFC        780
                 ********************************************* ***:**:*******

                 S2       S2-S3              S3                         S4
TRPM8_HUMAN
DEVRQWYVNGVNYFTDLWNVVMDTLGLFYFIAGIVFRLHSSNKSSLYSGRVIFCLDYIIFT       840
TRPM8_RAT
DEVRQWYMNGVNYFTDLWNVVMDTLGLFYFIAGIVFRLHSSNKSSLYSGRVIFCLDYIIFT       840
                 *******:****************************************************

                   S4                                  S5
TRPM8_HUMAN
LRLIHIFTVSRNLGPKIIMLQRMLIDVFFFLFLFAVWMVAFGVARQGILRQNEQRWRWIF        900
TRPM8_RAT
LRLIHIFTVSRNLGPKIIMLQRMLIDVFFFLFLFAVWMVAFGVARQGILRQNEQRWRWIF        900
                 ************************************************************

                   PH                                                          S6
TRPM8_HUMAN
RSVIYEPYLAMFGQVPSDVDGTTYDFAHCTFTGNESKPLCVELDEHNLPRFPEWITIPLV        960
TRPM8_RAT
RSVIYEPYLAMFGQVPSDVDSTTYDFSHCTFSGNESKPLCVELDEYNLPRFPEWITIPLV        960
                 ********************.*****:****:*************:**************

                    S6                                  TRP
TRPM8_HUMAN
CIYMLSTNILLVNLLVAMFGYTVGTVQENNDQVWKFQRYFLVQEYCSRLNIPFPFIVFAY        1020
TRPM8_RAT
CIYMLSTNILLVNLLVAMFGYTVGIVQENNDQVWKFQRYFLVQEYCNRLNIPFPFVVFAY        1020
                 ************************ *********************.********:****

TRPM8_HUMAN
FYMVVKKCFKCCCKEKNMESSVCCFKNEDNETLAWEGVMKENYLVKINTKANDTSEEMRH        1080
TRPM8_RAT
FYMVVKKCFKCCCKEKNTESSACCFRNEDNETLAWEGVMKENYLVKINTKANDNAEEMRH        1080
                 ***************** ***.***:***************************.:*****

TRPM8_HUMAN      RFRQLDTKLNDLKGLLKEIANKIK  1104
TRPM8_RAT        RFRQLDTKLNDLKGLLKEIANKIK  1104
                 ************************
```

Figure S2. Sequence Alignment of rat and human TRPM8. The asterisks denote absolute conserved residues, while colons and periods denote strong and weak similarity, respectively. Residues highlighted are critical for the sensitivity of TRPM8 to menthol (green), and icillin (yellow). Residue in blue or highlighted in blue are related to PIP2 binding. Boxes indicate TM secondary structures.

Table S4. Percentage of main docking solutions (and binding energy estimation in kcal/mol) obtained in molecular docking studies using Yasara. Truncated structure.

Subsite	Channel Location	TRPM8 without external loops % solutions (estimated binding energy, Kcal/mol)	
		**34**	**37**
1	Pore, external tower	_	_
2	Pore, high S3-S4, S6	20.2 (8.46)	17.1 (6.76)
3	Inner pore, S5S6, S5	16.4 (7.66)	14.9 (5.96)
4	Pore, internal mouth	9.2 (10.91)	23 (8.85)
5	Menthol bibding-site	0	0

Interactions at the different, most populated TRPM8 subsites 1-4

<table>
<tr><th colspan="2">Subsite 1 (Extracelular towers)</th></tr>
<tr><td>COMPD. 34 -- comp78_towers_62.pdb</td><td>Contactos a 4 A</td></tr>
<tr><td>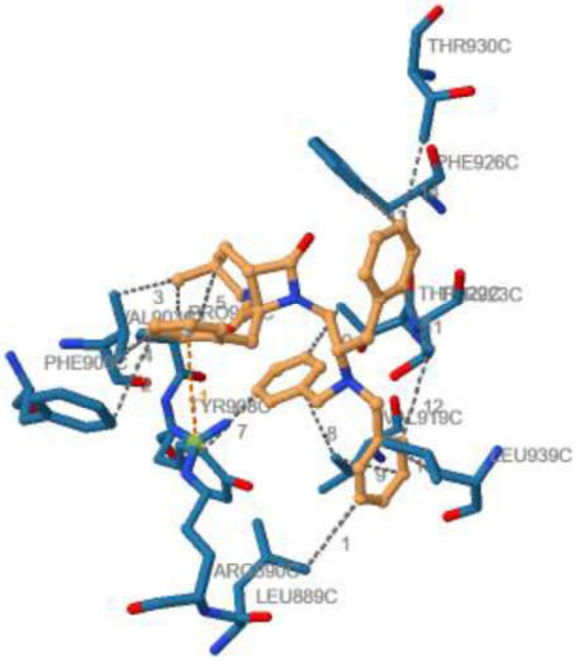
</td><td>Contact analysis
================
Residue LIG Z 1 :
Contacts to Residue GLU B 942 : 25
Contacts to Residue ARG B 950 : 22
Contacts to Residue ARG C 885 : 11
Contacts to Residue LEU C 889 : 20
Contacts to Residue ARG C 890 : 48
Contacts to Residue ILE C 899 : 7
Contacts to Residue PHE C 900 : 6
Contacts to Residue VAL C 903 : 73
Contacts to Residue PRO C 907 : 49
Contacts to Residue TYR C 908 : 16
Contacts to Residue MET C 911 : 21
Contacts to Residue PRO C 916 : 1
Contacts to Residue VAL C 919 : 68
Contacts to Residue ASP C 920 : 21
Contacts to Residue THR C 922 : 19
Contacts to Residue THR C 923 : 133
Contacts to Residue PHE C 926 : 25
Contacts to Residue SER C 927 : 12
Contacts to Residue THR C 930 : 17
Contacts to Residue LYS C 937 : 4
Contacts to Residue LEU C 939 : 69
Contacts to Residue CYS C 940 : 1
Contacts to Residue VAL C 941 : 1
23 contacts listed.</td></tr>
<tr><td>PLIP hydrophobic interactions</td><td></td></tr>
</table>

Hydrophobic Interactions

Index	Residue	AA	Distance	Ligand Atom	Protein Atom
1	889C	LEU	3.67	10562	6703
2	900C	PHE	3.99	10578	6818
3	903C	VAL	3.51	10560	6842
4	903C	VAL	3.16	10577	6843
5	907C	PRO	3.69	10559	6877
6	907C	PRO	3.56	10560	6878
7	908C	TYR	3.45	10569	6888
8	919C	VAL	3.71	10552	6970
9	919C	VAL	3.39	10564	6970
10	922C	THR	3.79	10567	6992
11	923C	THR	3.03	10571	6999
12	923C	THR	3.74	10565	6999
13	926C	PHE	3.57	10573	7027
14	930C	THR	3.39	10573	7059
15	939C	LEU	3.51	10550	7124

PLIP HBonds interaction // Pi- Stacking // Pi-Pi

π-Cation Interactions

Index	Residue	AA	Distance	Offset	Protein charged?	Ligand Group	Ligand Atoms
1	890C	ARG	4.11	1.33	✓	Aromatic	10553, 10576, 10577, 10578, 10579, 10580

Yasara Interactions

Hydrophobic Interaction Analysis
================================

Residue LEU C 889 :
to Residue LIG Z 1 : 1 interactions with strength 0.277
Residue PHE C 900 :
to Residue LIG Z 1 : 1 interactions with strength 0.971
Residue VAL C 903 :
to Residue LIG Z 1 : 1 interactions with strength 0.025
Residue PRO C 907 :
to Residue LIG Z 1 : 3 interactions with strength 0.580
Residue TYR C 908 :
to Residue LIG Z 1 : 1 interactions with strength 0.109
Residue VAL C 919 :
to Residue LIG Z 1 : 4 interactions with strength 2.352
Residue THR C 922 :
to Residue LIG Z 1 : 1 interactions with strength 0.507
Residue THR C 923 :
to Residue LIG Z 1 : 1 interactions with strength 0.423
Residue PHE C 926 :
to Residue LIG Z 1 : 2 interactions with strength 0.925
Residue LEU C 939 :
to Residue LIG Z 1 : 4 interactions with strength 1.085
Residue LIG Z 1 :
in Residue LIG Z 1 : 6 interactions with strength 1.549
11 interactions listed.

PiPi / CationPi / ionic

PiPi Interaction Analysis
=========================

Residue PHE C 900 :
to Residue LIG Z 1 : 2
interactions with strength 1.161
Residue PHE C 926 :
to Residue LIG Z 1 : 1
interactions with strength 0.480
2 interactions listed.

CationPi Interaction Analysis
=============================

0 interactions listed.

Ionic Interaction Analysis
==========================

0 interactions listed.

Hydrogen Bond Analysis

Hydrogen Bond Analysis<br>======================	

Subsite 1 (Extracelular towers)	
**COMPD. 35** – 79trans_towers_121.pdb	**Contactos a 4 A**
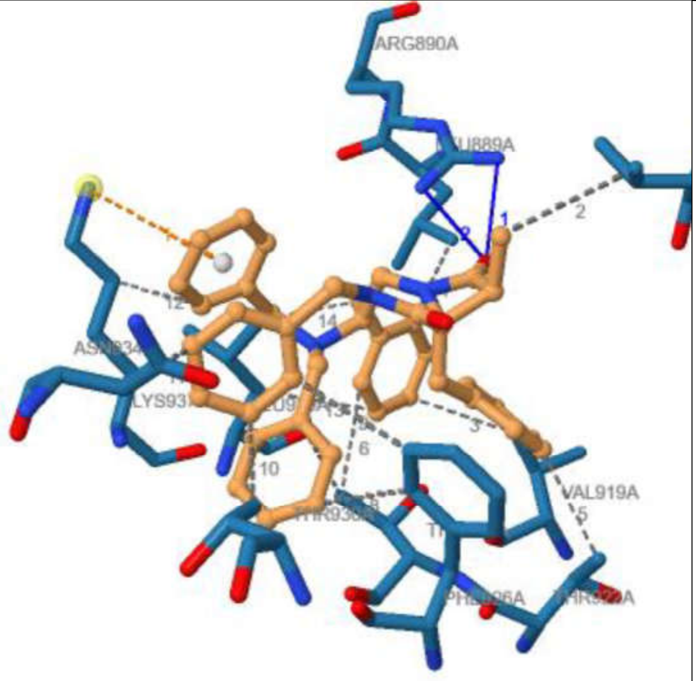 	Contact analysis<br>================<br>Residue LIG Z 1 :<br>Contacts to Residue ARG A 885 : 6<br>Contacts to Residue LEU A 889 : 27<br>Contacts to Residue ARG A 890 : 34<br>Contacts to Residue VAL A 903 : 19<br>Contacts to Residue TYR A 908 : 4<br>Contacts to Residue MET A 911 : 13<br>Contacts to Residue VAL A 919 : 40<br>Contacts to Residue ASP A 920 : 2<br>Contacts to Residue THR A 922 : 23<br>Contacts to Residue THR A 923 : 87<br>Contacts to Residue PHE A 926 : 54<br>Contacts to Residue SER A 927 : 35<br>Contacts to Residue THR A 930 : 25<br>Contacts to Residue ASN A 934 : 22<br>Contacts to Residue GLU A 935 : 28<br>Contacts to Residue SER A 936 : 30<br>Contacts to Residue LYS A 937 : 73<br>Contacts to Residue PRO A 938 : 1<br>Contacts to Residue LEU A 939 : 74<br>Contacts to Residue GLU D 942 : 1<br>20 contacts listed.
**PLIP hydrophobic interactions**	

Hydrophobic Interactions ····

Index	Residue	AA	Distance	Ligand Atom	Protein Atom
1	889A	LEU	3.40	10571	1436
2	903A	VAL	3.82	10544	1574
3	919A	VAL	3.23	10569	1702
4	919A	VAL	3.69	10575	1702
5	922A	THR	3.21	10573	1724
6	923A	THR	3.74	10567	1731
7	923A	THR	3.55	10550	1731
8	926A	PHE	3.59	10558	1759
9	926A	PHE	3.73	10577	1761
10	930A	THR	3.14	10578	1791
11	934A	ASN	3.26	10580	1817
12	937A	LYS	3.35	10562	1842
13	939A	LEU	3.54	10568	1856
14	939A	LEU	3.19	10547	1858

PLIP HBonds interaction // Pi- Stacking // Pi-Pi

Hydrogen Bonds

Index	Residue	AA	Distance H-A	Distance D-A	Donor Angle	Protein donor?	Side chain	Donor Atom	Acceptor Atom
1	890A	ARG	2.60	3.45	145.85	✓	✓	1446 [Ng+]	10582 [O2]
2	890A	ARG	2.30	3.23	157.68	✓	✓	1447 [Ng+]	10582 [O2]

π-Cation Interactions ····

Index	Residue	AA	Distance	Offset	Protein charged?	Ligand Group	Ligand Atoms
1	937A	LYS	4.82	1.11	✓	Aromatic	10552, 10562, 10563, 10564, 10565, 10566

Yasara Interactions

Hydrophobic Interaction Analysis
================================
Residue VAL A 903 :
to Residue LIG Z 1 : 1 interactions with strength 0.644
Residue MET A 911 :
to Residue LIG Z 1 : 1 interactions with strength 0.585
Residue VAL A 919 :
to Residue LIG Z 1 : 2 interactions with strength 0.997
Residue THR A 923 :
to Residue LIG Z 1 : 8 interactions with strength 2.953
Residue PHE A 926 :
to Residue LIG Z 1 : 3 interactions with strength 1.543
Residue LYS A 937 :
to Residue LIG Z 1 : 2 interactions with strength 0.277
Residue LEU A 939 :
to Residue LIG Z 1 : 2 interactions with strength 0.178
Residue LIG Z 1 :
in Residue LIG Z 1 : 9 interactions with strength 5.041
8 interactions listed.

PiPi / CationPi / ionic

PiPi Interaction Analysis
=========================
Residue PHE A 926 :
to Residue LIG Z 1 : 1 interactions with strength 0.419
Residue LIG Z 1 :
in Residue LIG Z 1: 12 interactions with strength 9.269
2 interactions listed.

CationPi Interaction Analysis
=============================
Residue LYS A 937 :
to Residue LIG Z 1 : 2 interactions with strength 0.998
1 interaction listed.

Ionic Interaction Analysis

	==========================<br>0 interactions listed.
**Hydrogen Bond Analysis**	
Hydrogen Bond Analysis<br>======================<br>Residue LIG Z 1 :<br>Atom O LIG 1 Z accepts a bond from NH2 ARG 890 A, O - H distance is 2.29 A, bond energy is 3.29 kcal/mol.<br>1 hydrogen bonds better than 1.49 kcal/mol, 1 accepted, 0 donated. Total hydrogen bond energy is 3.29 kcal/mol.	

**Subsite 1 (Extracelular towers)**	
**COMPD. 37** -- comp55_towers_6.pdb	**Contactos a 4 A**
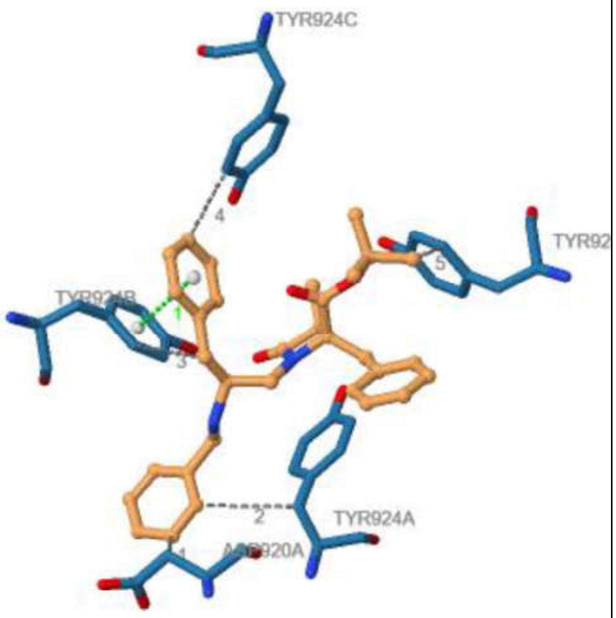 	Contact analysis<br>================<br>Residue LIG Z 1 :<br>Contacts to Residue ASP A 920 : 23<br>Contacts to Residue SER A 921 : 24<br>Contacts to Residue THR A 923 : 1<br>Contacts to Residue TYR A 924 : 88<br>Contacts to Residue ASP A 925 : 23<br>Contacts to Residue PRO A 938 : 8<br>Contacts to Residue LEU A 939 : 3<br>Contacts to Residue CYS A 940 : 7<br>Contacts to Residue ASP B 920 : 1<br>Contacts to Residue SER B 921 : 25<br>Contacts to Residue TYR B 924 : 81<br>Contacts to Residue ASP B 925 : 38<br>Contacts to Residue HIS B 928 : 19<br>Contacts to Residue SER C 921 : 4<br>Contacts to Residue TYR C 924 : 17<br>Contacts to Residue ASP C 925 : 6<br>Contacts to Residue SER D 917 : 12

Contacts to Residue ASP D 920 : 9
Contacts to Residue SER D 921 : 62
Contacts to Residue TYR D 924 : 47
Contacts to Residue ASP D 925 : 14
21 contacts listed.

PLIP hydrophobic interactions

Hydrophobic Interactions

Index	Residue	AA	Distance	Ligand Atom	Protein Atom
1	920A	ASP	3.64	10557	1708
2	924A	TYR	3.63	10556	1736
3	924B	TYR	3.53	10546	4373
4	924C	TYR	3.73	10563	7009
5	924D	TYR	3.24	10571	9641

PLIP HBonds interaction // Pi- Stacking // Pi-Pi

π-Stacking

Index	Residue	AA	Distance	Angle	Offset	Stacking Type	Ligand Atoms
1	924B	TYR	3.98	26.13	1.48	P	10548, 10561, 10562, 10563, 10564, 10565

Yasara Interactions

Hydrophobic Interaction Analysis
================================

Residue ASP A 920 :
to Residue LIG Z 1 : 1 interactions with strength 0.278
Residue TYR A 924 :
to Residue LIG Z 1 : 6 interactions with strength 1.916
Residue SER B 921 :
to Residue LIG Z 1 : 1 interactions with strength 0.310
Residue TYR B 924 :
to Residue LIG Z 1 : 5 interactions with strength 1.193
Residue HIS B 928 :
to Residue LIG Z 1 : 1 interactions with strength 0.057
Residue TYR C 924 :
to Residue LIG Z 1 : 2 interactions with strength 1.386
Residue SER D 921 :
to Residue LIG Z 1 : 1 interactions with strength 0.466
Residue LIG Z 1 :
in Residue LIG Z 1 : 4 interactions with strength 1.237
8 interactions listed.

PiPi / CationPi / ionic

PiPi Interaction Analysis
=========================

Residue TYR A 924 :
to Residue LIG Z 1 : 2 interactions with strength 0.594
Residue TYR B 924 :
to Residue LIG Z 1 : 1 interactions with strength 0.156
Residue HIS B 928 :
to Residue LIG Z 1 : 1 interactions with strength 0.297
Residue TYR D 924 :
to Residue LIG Z 1 : 1 interactions with strength 0.075
4 interactions listed.

CationPi Interaction Analysis
============================

0 interactions listed.

Ionic Interaction Analysis
==========================

0 interactions listed.

Hydrogen Bond Analysis

Hydrogen Bond Analysis<br>======================<br>Residue TYR B 924 :<br>Atom OH TYR 924 B donates a bond to O LIG 1 Z, H - O distance is 2.12 A, bond energy is 22.48 kJ/mol.<br>1 hydrogen bonds better than 6.25 kJ/mol, 0 accepted, 1 donated. Total hydrogen bond energy is 22.48 kJ/mol.<br>Residue ASP B 925 :<br>Atom OD1 ASP 925 B accepts a bond from N1 LIG 1 Z, O - H distance is 1.83 A, bond energy is 21.88 kJ/mol.<br>1 hydrogen bonds better than 6.25 kJ/mol, 1 accepted, 0 donated. Total hydrogen bond energy is 21.88 kJ/mol.	

**Subsite 2 (S3,S4, S6)**	
**COMD. 34** -- comp78_S3S4S6_73.pdb	**Contactos a 4 A**
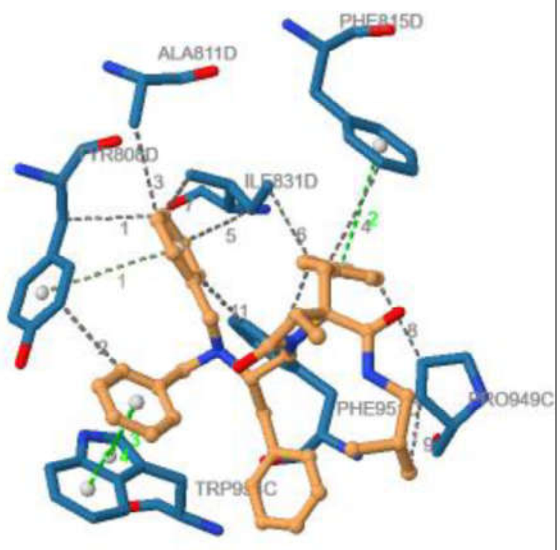	Contact analysis<br>================<br>Residue LIG Z 1 :<br>Contacts to Residue PRO C 949 : 95<br>Contacts to Residue ARG C 950 : 35<br>Contacts to Residue PHE C 951 : 119<br>Contacts to Residue GLU C 953 : 13<br>Contacts to Residue TRP C 954 : 97<br>Contacts to Residue PHE D 807 : 1<br>Contacts to Residue TYR D 808 : 57<br>Contacts to Residue ALA D 811 : 18<br>Contacts to Residue GLY D 812 : 2<br>Contacts to Residue PHE D 815 : 14<br>Contacts to Residue SER D 827 : 28<br>Contacts to Residue VAL D 830 : 2<br>Contacts to Residue ILE D 831 : 63<br>Contacts to Residue TRP D 898 : 13<br>14 contacts listed.
**PLIP hydrophobic interactions**	

Index	Residue	AA	Distance	Ligand Atom	Protein Atom
1	808D	TYR	3.89	10568	8666
2	808D	TYR	3.30	10565	8671
3	811D	ALA	3.32	10567	8697
4	815D	PHE	4.00	10541	8725
5	831D	ILE	3.46	10569	8851
6	831D	ILE	3.17	10579	8853
7	831D	ILE	3.83	10568	8852
8	949C	PRO	3.31	10577	7208
9	949C	PRO	3.34	10560	7207
10	951C	PHE	3.38	10579	7227
11	951C	PHE	3.12	10570	7229

PLIP HBonds interaction // Pi- Stacking // Pi-Pi

π-Stacking

Index	Residue	AA	Distance	Angle	Offset	Stacking Type	Ligand Atoms
1	808D	Amino	Distance between ring centers	[illegible]	[illegible]	[illegible]	10552, 10566, 10567, 10568, 10569, 10570
2	815D	PHE	4.44	26.91	0.93	P	10553, 10576, 10577, 10578, 10579, 10580
3	954C	TRP	3.85	6.63	1.32	P	10550, 10561, 10562, 10563, 10564, 10565
4	954C	TRP	3.63	5.85	0.70	P	10550, 10561, 10562, 10563, 10564, 10565

Yasara Interactions

Hydrophobic Interaction Analysis
================================

Residue PHE C 951 :
to Residue LIG Z 1 : 3 interactions with strength 0.798
Residue TRP C 954 :
to Residue LIG Z 1 : 16 interactions with strength 8.442
Residue TYR D 808 :
to Residue LIG Z 1 : 3 interactions with strength 1.328
Residue PHE D 815 :
to Residue LIG Z 1 : 3 interactions with strength 2.696
Residue ILE D 831 :
to Residue LIG Z 1 : 4 interactions with strength 1.093
Residue LIG Z 1 :
in Residue LIG Z 1 : 8 interactions with strength 5.247
6 interactions listed.

PiPi / CationPi / ionic

PiPi Interaction Analysis
=========================

Residue PHE C 951 :
to Residue LIG Z 1 : 1
interactions with strength 1.000
Residue PHE D 807 :
to Residue LIG Z 1 : 1
interactions with strength 0.069
Residue TYR D 808 :
to Residue LIG Z 1 : 2
interactions with strength 1.444
Residue LIG Z 1 :
in Residue LIG Z 1 : 2
interactions with strength 1.335
4 interactions listed.

CationPi Interaction Analysis
===========================
==

0 interactions listed.

Ionic Interaction Analysis
==========================

	0 interactions listed.
**Hydrogen Bond Analysis**	
Hydrogen Bond Analysis<br>======================	

**Subsite 2 (S3,S4, S6)**	
**COMPD. 35**– 79trans_S3S4S6_23.pdb	**Contactos a 4 A**
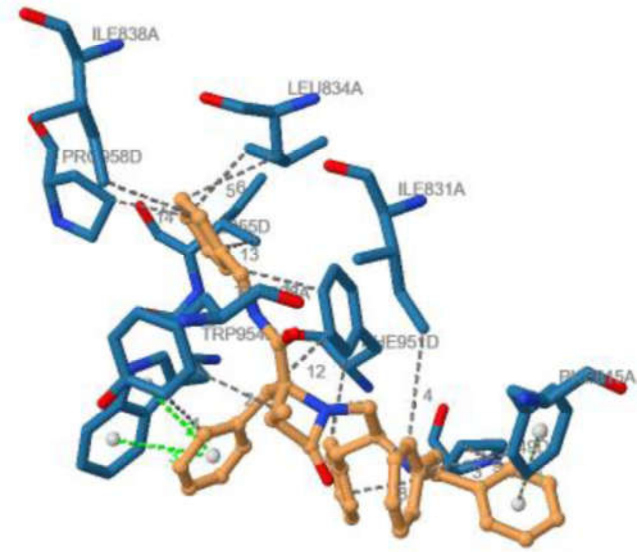	Contact analysis<br>================<br>Residue LIG Z 1 :<br>Contacts to Residue PHE A 807 :<br>2<br>Contacts to Residue TYR A 808 :<br>99<br>Contacts to Residue ALA A 811 :<br>32<br>Contacts to Residue GLY A 812 :<br>1<br>Contacts to Residue PHE A 815 :<br>79<br>Contacts to Residue SER A 827 :<br>2<br>Contacts to Residue ILE A 831 :<br>54<br>Contacts to Residue LEU A 834 :<br>59<br>Contacts to Residue ASP A 835 :<br>23<br>Contacts to Residue ILE A 838 :<br>21<br>Contacts to Residue LEU D 948 :<br>4<br>Contacts to Residue PRO D 949 :<br>71<br>Contacts to Residue ARG D 950 :<br>17<br>Contacts to Residue PHE D 951 :<br>125<br>Contacts to Residue TRP D 954 :<br>42<br>Contacts to Residue ILE D 955 :<br>31<br>Contacts to Residue PRO D 958 :<br>29<br>17 contacts listed.
**PLIP hydrophobic interactions**	

Hydrophobic Interactions

Index	Residue	AA	Distance	Ligand Atom	Protein Atom
1	808A	TYR	3.06	10576	769
2	808A	TYR	3.22	10544	767
3	815A	PHE	3.24	10552	821
4	831A	ILE	3.77	10566	951
5	834A	LEU	3.34	10579	975
6	834A	LEU	3.43	10578	973
7	838A	ILE	3.59	10577	1012
8	949D	PRO	3.48	10569	9841
9	949D	PRO	3.22	10561	9842
10	951D	PHE	3.41	10571	9861
11	951D	PHE	3.31	10581	9865
12	951D	PHE	2.97	10543	9863
13	955D	ILE	3.22	10580	9902
14	958D	PRO	3.01	10579	9924

PLIP HBonds interaction // Pi- Stacking // Pi-Pi

π-Stacking

Index	Residue	AA	Distance	Angle	Offset	Stacking Type	Ligand Atoms
1	815A	PHE	4.49	69.79	0.10	T	10550, 10557, 10558, 10559, 10560, 10561
2	954D	TRP	3.83	10.20	0.09	P	10553, 10572, 10573, 10574, 10575, 10576
3	954D	TRP	4.12	9.58	1.65	P	10553, 10572, 10573, 10574, 10575, 10576

Yasara Interactions

Hydrophobic Interaction Analysis
================================
Residue TYR A 808 :
 to Residue LIG Z 1 : 1 interactions with strength 0.077
Residue ALA A 811 :
 to Residue LIG Z 1 : 1 interactions with strength 0.486
Residue PHE A 815 :
 to Residue LIG Z 1 : 4 interactions with strength 2.963
Residue ILE A 831 :
 to Residue LIG Z 1 : 2 interactions with strength 0.487
Residue LEU A 834 :
 to Residue LIG Z 1 : 1 interactions with strength 0.687
Residue ILE A 838 :
 to Residue LIG Z 1 : 2 interactions with strength 0.362
Residue PRO D 949 :
 to Residue LIG Z 1 : 2 interactions with strength 0.237
Residue PHE D 951 :
 to Residue LIG Z 1 : 4 interactions with strength 1.970
Residue TRP D 954 :
 to Residue LIG Z 1 : 8 interactions with strength 6.483
Residue PRO D 958 :
 to Residue LIG Z 1 : 1 interactions with strength 0.156
Residue LIG Z 1 :
 In Residue LIG Z 1 : 4 interactions with strength 1.758

PiPi / CationPi / ionic

PiPi Interaction Analysis
=========================
Residue PHE A 815 :
 to Residue LIG Z 1 : 4 interactions with strength 6.000
Residue HIS A 818 :
 to Residue LIG Z 1 : 1 interactions with strength 1.000
Residue PHE D 951 :
 to Residue LIG Z 1 : 1 interactions with strength 0.067
Residue TRP D 954 :
 to Residue LIG Z 1 : 1 interactions with strength 0.028
4 interactions listed.

CationPi Interaction Analysis
===========================
=

0 interactions listed.

11 interactions listed.	Ionic Interaction Analysis<br>==========================<br><br>0 interactions listed.
**Hydrogen Bond Analysis**	
Hydrogen Bond Analysis<br>======================	

**Subsite 2 (S3,S4, S6)**	
**COMPD. 37** -- comp55_S3S4S6_50.pdb	**Contactos a 4 A**
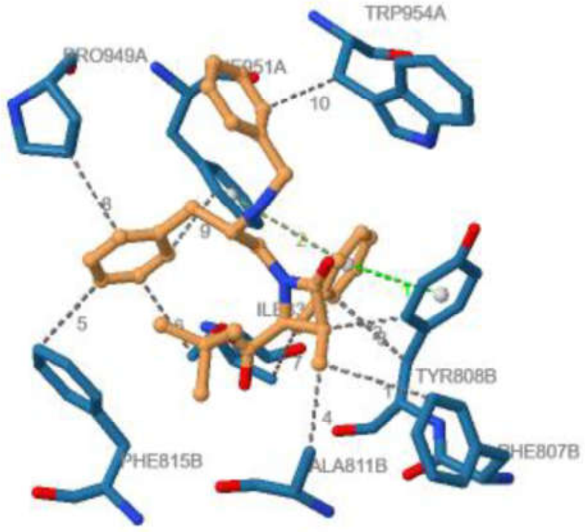	Contact analysis<br>================<br>Residue LIG Z 1 :<br>Contacts to Residue PRO A 949 : 29<br>Contacts to Residue ARG A 950 : 13<br>Contacts to Residue PHE A 951 : 111<br>Contacts to Residue GLU A 953 : 3<br>Contacts to Residue TRP A 954 : 38<br>Contacts to Residue ILE A 955 : 1<br>Contacts to Residue PRO A 958 : 1<br>Contacts to Residue PHE B 807 : 10<br>Contacts to Residue TYR B 808 : 87<br>Contacts to Residue ALA B 811 : 52<br>Contacts to Residue GLY B 812 : 4<br>Contacts to Residue PHE B 815 : 59<br>Contacts to Residue SER B 827 : 22<br>Contacts to Residue ILE B 831 : 97<br>Contacts to Residue LEU B 834 : 10<br>Contacts to Residue ASP B 835 : 13<br>16 contacts listed.
**PLIP hydrophobic interactions**	

Hydrophobic Interactions

Index	Residue	AA	Distance	Ligand Atom	Protein Atom
1	807B	PHE	3.87	10542	3392
2	808B	TYR	2.94	10537	3401
3	808B	TYR	3.53	10570	3398
4	811B	ALA	2.95	10542	3429
5	815B	PHE	3.31	10563	3459
6	831B	ILE	3.35	10562	3585
7	831B	ILE	3.25	10541	3584
8	949A	PRO	3.41	10565	1940
9	951A	PHE	3.09	10561	1959
10	954A	TRP	3.28	10560	1984

PLIP HBonds interaction // Pi- Stacking // Pi-Pi

π-Stacking

Index	Residue	AA	Distance	Angle	Offset	Stacking Type	Ligand Atoms
1	808B	TYR	3.87	23.24	1.59	P	10551, 10566, 10567, 10568, 10569, 10570
2	951A	PHE	4.64	70.58	0.40	T	10551, 10566, 10567, 10568, 10569, 10570

**Yasara Interactions**	**PiPi / CationPi / ionic**
Hydrophobic Interaction Analysis<br>================================<br><br>Residue PRO A 949 :<br>to Residue LIG Z 1 : 1 interactions with strength 0.222<br>Residue PHE A 951 :<br>to Residue LIG Z 1 : 10 interactions with strength 5.025<br>Residue TRP A 954 :<br>to Residue LIG Z 1 : 1 interactions with strength 0.364<br>Residue PHE B 807 :<br>to Residue LIG Z 1 : 1 interactions with strength 0.652<br>Residue TYR B 808 :<br>to Residue LIG Z 1 : 10 interactions with strength 3.750<br>Residue PHE B 815 :<br>to Residue LIG Z 1 : 2 interactions with strength 0.107<br>Residue ILE B 831 :<br>to Residue LIG Z 1 : 2 interactions with strength 0.200<br>Residue LIG Z 1 :<br>in Residue LIG Z 1 : 2 interactions with strength 0.820<br>8 interactions listed.	PiPi Interaction Analysis<br>=========================<br><br>Residue PHE A 951 :<br>to Residue LIG Z 1 : 7 interactions with strength 5.358<br>Residue TRP A 954 :<br>to Residue LIG Z 1 : 1 interactions with strength 0.323<br>Residue TYR B 808 :<br>to Residue LIG Z 1 : 1 interactions with strength 0.571<br>Residue PHE B 815 :<br>to Residue LIG Z 1 : 1 interactions with strength 0.559<br>4 interactions listed.<br><br>CationPi Interaction Analysis<br>=============================<br><br>0 interactions listed.<br><br>Ionic Interaction Analysis<br>==========================<br><br>0 interactions listed.
**Hydrogen Bond Analysis**	
Hydrogen Bond Analysis	

=====================
Residue LIG Z 1 :
Atom O LIG 1 Z accepts a bond from N1 LIG 1 Z, O - H distance is 2.12 A, bond energy is 7.50 kJ/mol.
Atom N1 LIG 1 Z donates a bond to O LIG 1 Z, H - O distance is 2.12 A, bond energy is 7.50 kJ/mol.
2 hydrogen bonds better than 6.25 kJ/mol, 1 accepted, 1 donated. Total hydrogen bond energy is 15.00 kJ/mol.

Subsite 3 (S5,S6)

COMPD. 34 -- comp78_S5S6_41.pdb

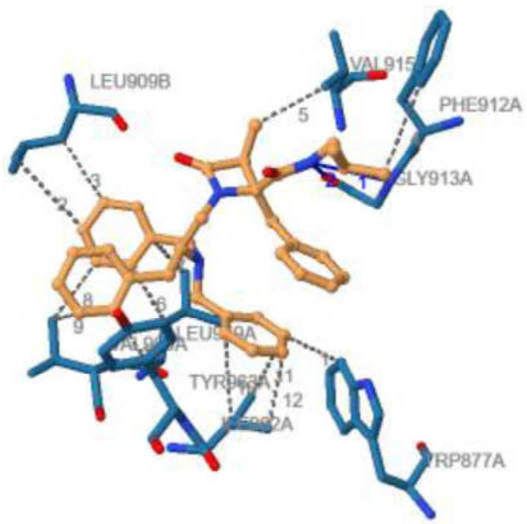

Contactos a 4 A

Contact analysis
================
Residue LIG Z 1 :
Contacts to Residue PHE A 874 : 8
Contacts to Residue TRP A 877 : 27
Contacts to Residue PHE A 912 : 55
Contacts to Residue GLY A 913 : 71
Contacts to Residue GLN A 914 : 21
Contacts to Residue VAL A 915 : 36
Contacts to Residue THR A 956 : 26
Contacts to Residue LEU A 959 : 94
Contacts to Residue VAL A 960 : 87
Contacts to Residue ILE A 962 : 53
Contacts to Residue TYR A 963 : 64
Contacts to Residue LEU B 871 : 16
Contacts to Residue PHE B 874 : 9
Contacts to Residue LEU B 909 : 41
Contacts to Residue ALA B 910 : 24
Contacts to Residue PHE B 912 : 1
16 contacts listed.

PLIP hydrophobic interactions

Hydrophobic Interactions

Index	Residue	AA	Distance	Ligand Atom	Protein Atom
1	877A	TRP	3.45	10564	1347
2	909B	LEU	3.90	10568	4265
3	909B	LEU	3.47	10567	4262
4	912A	PHE	3.69	10559	1652
5	915A	VAL	3.32	10542	1675
6	959A	LEU	3.25	10570	2028
7	959A	LEU	3.59	10552	2030
8	960A	VAL	3.13	10569	2038
9	960A	VAL	3.19	10574	2038
10	962A	ILE	3.88	10561	2049
11	962A	ILE	3.63	10564	2052
12	962A	ILE	3.14	10563	2051
13	963A	TYR	3.15	10575	2057

PLIP HBonds interaction // Pi- Stacking // Pi-Pi

Hydrogen Bonds

Index	Residue	AA	Distance H-A	Distance D-A	Donor Angle	Protein donor?	Side chain	Donor Atom	Acceptor Atom
1	913A	GLY	3.06	3.45	105.17	✔	×	1656 [Nam]	10555 [Nam]
2	913A	GLY	1.81	2.79	174.34	×	×	10555 [Nam]	1659 [O2]

Yasara Interactions	PiPi / CationPi / ionic
Hydrophobic Interaction Analysis<br>================================<br><br>Residue TRP A 877 :<br>to Residue LIG Z 1 : 1 interactions with strength 0.122<br>Residue PHE A 912 :<br>to Residue LIG Z 1 : 2 interactions with strength 0.406<br>Residue LEU A 959 :<br>to Residue LIG Z 1 : 3 interactions with strength 0.956<br>Residue ILE A 962 :<br>to Residue LIG Z 1 : 4 interactions with strength 1.374<br>Residue TYR A 963 :<br>to Residue LIG Z 1 : 3 interactions with strength 1.841<br>Residue LEU B 909 :<br>to Residue LIG Z 1 : 2 interactions with strength 1.471<br>Residue LIG Z 1 :<br>in Residue LIG Z 1 : 18 interactions with strength 8.487<br>7 interactions listed.	PiPi Interaction Analysis<br>=========================<br><br>Residue TRP A 877 :<br>to Residue LIG Z 1 : 1 interactions with strength 0.051<br>Residue LIG Z 1 :<br>in Residue LIG Z 1 : 4 interactions with strength 1.477<br>2 interactions listed.<br><br>CationPi Interaction Analysis<br>============================<br>=<br><br>0 interactions listed.<br><br>Ionic Interaction Analysis<br>==========================<br><br>0 interactions listed.
**Hydrogen Bond Analysis**	
Hydrogen Bond Analysis<br>======================<br>Residue GLY A 913 :<br>Atom O GLY 913 A accepts a bond from N LIG 1 Z, O -H distance is 1.77 A, bond energy is 25.00 kJ/mol.<br>1 hydrogen bonds better than 6.25 kJ/mol, 1 accepted, 0 donated. Total hydrogen bond energy is 25.00 kJ/mol.	

Subsite 3 (S5,S6)	
**COMPD. 35** – 79trans_S5S6_35.pdb	**Contactos a 4 A**

<table>
<tr>
<td>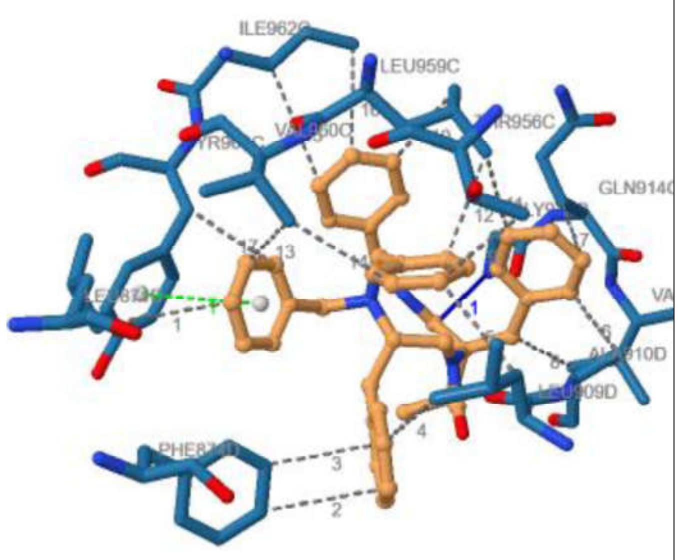
</td>
<td>Contact analysis
================
Residue LIG Z 1 :
Contacts to Residue TRP C 877 : 1
Contacts to Residue GLY C 913 : 16
Contacts to Residue GLN C 914 : 62
Contacts to Residue VAL C 915 : 27
Contacts to Residue PRO C 916 : 2
Contacts to Residue THR C 956 : 34
Contacts to Residue LEU C 959 : 102
Contacts to Residue VAL C 960 : 63
Contacts to Residue ILE C 962 : 30
Contacts to Residue TYR C 963 : 40
Contacts to Residue LEU D 871 : 16
Contacts to Residue PHE D 874 : 27
Contacts to Residue MET D 878 : 22
Contacts to Residue LEU D 909 : 66
Contacts to Residue ALA D 910 : 59
Contacts to Residue MET D 911 : 3
Contacts to Residue PHE D 912 : 10
Contacts to Residue GLY D 913 : 3
18 contacts listed.</td>
</tr>
<tr>
<td>PLIP hydrophobic interactions</td>
<td></td>
</tr>
</table>

Hydrophobic Interactions ····

Index	Residue	AA	Distance	Ligand Atom	Protein Atom
1	871D	LEU	3.94	10559	9195
2	874D	PHE	3.79	10569	9225
3	874D	PHE	3.56	10570	9224
4	909D	LEU	3.59	10570	9533
5	909D	LEU	3.53	10564	9530
6	910D	ALA	3.23	10576	9538
7	914C	GLN	3.24	10575	6932
8	915C	VAL	3.50	10543	6943
9	956C	THR	3.35	10564	7276
10	959C	LEU	3.41	10578	7299
11	959C	LEU	3.33	10573	7298
12	959C	LEU	3.37	10566	7298
13	960C	VAL	3.32	10560	7306
14	960C	VAL	3.50	10563	7306
15	962C	ILE	3.97	10580	7317
16	962C	ILE	3.45	10579	7320
17	963C	TYR	3.35	10561	7325

PLIP HBonds interaction // Pi- Stacking // Pi-Pi

Hydrogen Bonds —

Index	Residue	AA	Distance H-A	Distance D-A	Donor Angle	Protein donor?	Side chain	Donor Atom	Acceptor Atom
1	913C	GLY	3.43	3.89	110.81	✔	✖	6924 [Nam]	10583 [O2]

π-Stacking ···· ····

Index	Residue	AA	Distance	Angle	Offset	Stacking Type	Ligand Atoms
1	963C	TYR	4.36	28.54	0.43	P	10550, 10557, 10558, 10559, 10560, 10561

Yasara Interactions	PiPi / CationPi / ionic
Hydrophobic Interaction Analysis<br>================================<br>Residue LEU C 959 :<br>to Residue LIG Z 1 : 2 interactions with strength 1.635<br>Residue VAL C 960 :<br>to Residue LIG Z 1 : 1 interactions with strength 0.654<br>Residue ILE C 962 :<br>to Residue LIG Z 1 : 2 interactions with strength 1.284<br>Residue TYR C 963 :<br>to Residue LIG Z 1 : 2 interactions with strength 1.605<br>Residue LEU D 871 :<br>to Residue LIG Z 1 : 1 interactions with strength 0.790<br>Residue PHE D 874 :<br>to Residue LIG Z 1 : 3 interactions with strength 1.884<br>Residue LEU D 909 :	PiPi Interaction Analysis<br>=========================<br>Residue TYR C 963 :<br>to Residue LIG Z 1 : 2 interactions with strength 0.832<br>Residue PHE D 874 :<br>to Residue LIG Z 1 : 3 interactions with strength 1.564<br>Residue LIG Z 1 :<br>in Residue LIG Z 1 : 3 interactions with strength 4.946<br>3 interactions listed.

to Residue LIG Z 1 : 3 interactions with strength 0.873<br>Residue LIG Z 1 :<br>in Residue LIG Z 1 : 9 interactions with strength 5.281<br>8 interactions listed.	CationPi Interaction Analysis<br>=========================<br>===<br><br>0 interactions listed.<br><br><br>Ionic Interaction Analysis<br>=========================<br><br>0 interactions listed.
**Hydrogen Bond Analysis**	
Hydrogen Bond Analysis<br>=====================<br>Residue LIG Z 1 :<br>Atom N LIG 1 Z accepts a bond from N LIG 1 Z, N - H distance is 2.07 A, bond energy is 4.76 kcal/mol.<br>Atom N LIG 1 Z donates a bond to N LIG 1 Z, H - N distance is 2.07 A, bond energy is 4.76 kcal/mol.<br>2 hydrogen bonds better than 1.49 kcal/mol, 1 accepted, 1 donated. Total hydrogen bond energy is 9.52 kcal/mol.	

**Subsite 3 (S5,S6)**	
**COMPD. 37** -- comp55_S5S6_41.pdb	**Contactos a 4 A**
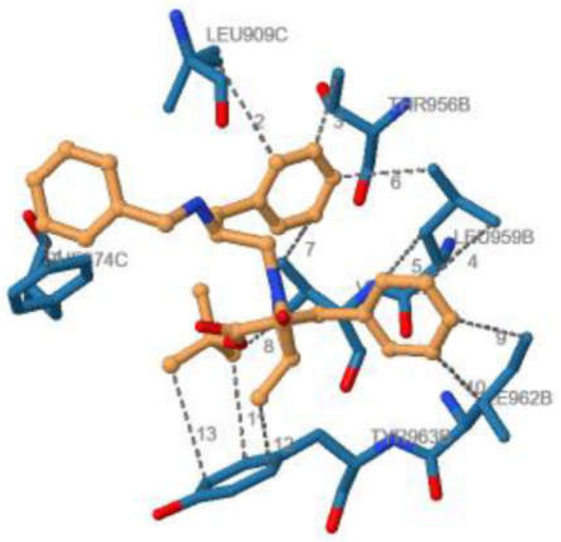	Contact analysis<br>================<br>Residue LIG Z 1 :<br>Contacts to Residue TRP B 877 : 5<br>Contacts to Residue GLN B 914 : 7<br>Contacts to Residue ILE B 955 : 1<br>Contacts to Residue THR B 956 : 48<br>Contacts to Residue LEU B 959 : 89<br>Contacts to Residue VAL B 960 : 94<br>Contacts to Residue ILE B 962 : 35<br>Contacts to Residue TYR B 963 : 80<br>Contacts to Residue LEU C 871 : 60<br>Contacts to Residue PHE C 874 : 42<br>Contacts to Residue TRP C 877 : 2<br>Contacts to Residue MET C 878 : 21<br>Contacts to Residue PHE C 881 : 1<br>Contacts to Residue TYR C 908 : 4<br>Contacts to Residue LEU C 909 : 57<br>Contacts to Residue ALA C 910 : 6<br>Contacts to Residue MET C 911 : 8<br>Contacts to Residue PHE C 912 : 3<br>Contacts to Residue GLY C 913 : 23<br>19 contacts listed.

PLIP hydrophobic interactions

Hydrophobic Interactions ····

Index	Residue	AA	Distance	Ligand Atom	Protein Atom
1	874C	PHE	3.57	10557	6591
2	909C	LEU	3.62	10565	6896
3	956B	THR	3.30	10564	4642
4	959B	LEU	3.57	10567	4665
5	959B	LEU	3.69	10566	4662
6	959B	LEU	3.30	10563	4664
7	960B	VAL	3.24	10562	4672
8	960B	VAL	3.36	10571	4671
9	962B	ILE	3.56	10568	4686
10	962B	ILE	3.47	10569	4683
11	963B	TYR	3.54	10571	4694
12	963B	TYR	3.37	10542	4693
13	963B	TYR	3.90	10572	4696

PLIP HBonds interaction // Pi- Stacking // Pi-Pi

Yasara Interactions	PiPi / CationPi / ionic
Hydrophobic Interaction Analysis<br>================================<br><br>Residue LEU B 959 :<br>to Residue LIG Z 1 : 7 interactions with strength 3.292<br>Residue VAL B 960 :<br>to Residue LIG Z 1 : 2 interactions with strength 0.158<br>Residue ILE B 962 :<br>to Residue LIG Z 1 : 2 interactions with strength 0.595<br>Residue TYR B 963 :<br>to Residue LIG Z 1 : 5 interactions with strength 2.315<br>Residue LEU C 871 :<br>to Residue LIG Z 1 : 1 interactions with strength 0.591<br>Residue PHE C 874 :<br>to Residue LIG Z 1 : 4 interactions with strength 2.041<br>Residue MET C 878 :<br>to Residue LIG Z 1 : 1 interactions with strength 0.078<br>Residue LEU C 909 :<br>to Residue LIG Z 1 : 1 interactions with strength 0.241<br>Residue GLY C 913 :<br>to Residue LIG Z 1 : 2 interactions with strength 0.696	PiPi Interaction Analysis<br>=========================<br><br>Residue LIG Z 1 :<br>in Residue LIG Z 1 : 4 interactions with strength 2.648<br>1 interaction listed.<br><br>CationPi Interaction Analysis<br>============================<br>=<br><br>0 interactions listed.<br><br>Ionic Interaction Analysis<br>==========================<br><br>0 interactions listed.

Residue LIG Z 1 :<br>in Residue LIG Z 1 : 3 interactions with strength 0.758<br>10 interactions listed.	
**Hydrogen Bond Analysis**	
Hydrogen Bond Analysis<br>======================<br>Residue LEU C 909 :<br>Atom O LEU 909 C accepts a bond from N1 LIG 1 Z, O -H distance is 1.94 A, bond energy is 6.85 kJ/mol.<br>1 hydrogen bonds better than 6.25 kJ/mol, 1 accepted, 0 donated. Total hydrogen bond energy is 6.85 kJ/mol.	

**INNER MOUTH-PORE**	
**COMPD. 34** -- comp55_mouthpore_22.pdb	**Contactos a 4 A**
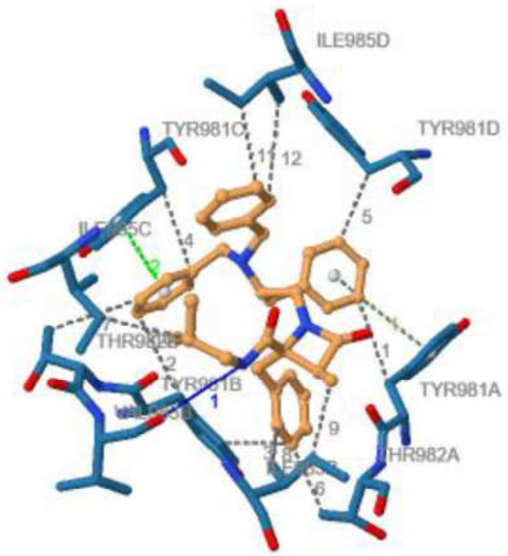	Contact analysis<br>================<br>Residue LIG Z 1 :<br>Contacts to Residue MET A 978 : 30<br>Contacts to Residue PHE A 979 : 8<br>Contacts to Residue TYR A 981 : 74<br>Contacts to Residue THR A 982 : 42<br>Contacts to Residue VAL A 983 : 8<br>Contacts to Residue GLY A 984 : 4<br>Contacts to Residue ILE A 985 : 1<br>Contacts to Residue MET B 978 : 23<br>Contacts to Residue PHE B 979 : 7<br>Contacts to Residue TYR B 981 : 68<br>Contacts to Residue THR B 982 : 33<br>Contacts to Residue VAL B 983 : 16<br>Contacts to Residue GLY B 984 : 18<br>Contacts to Residue ILE B 985 : 66<br>Contacts to Residue VAL B 986 : 1<br>Contacts to Residue TRP B 994 : 1<br>Contacts to Residue MET C 978 : 6<br>Contacts to Residue TYR C 981 : 62<br>Contacts to Residue VAL C 983 : 7<br>Contacts to Residue GLY C 984 : 10<br>Contacts to Residue ILE C 985 : 75<br>Contacts to Residue VAL C 986 : 4<br>Contacts to Residue MET D 978 : 32<br>Contacts to Residue PHE D 979 : 1<br>Contacts to Residue TYR D 981 : 25<br>Contacts to Residue GLY D 984 : 1<br>Contacts to Residue ILE D 985 : 33<br>27 contacts listed.
**PLIP hydrophobic interactions**	

Index	Residue	AA	Distance	Ligand Atom	Protein Atom
1	981A	TYR	3.80	10572	2196
2	981B	TYR	3.75	10568	4830
3	981B	TYR	3.28	10577	4833
4	981C	TYR	3.53	10552	7464
5	981D	TYR	3.18	10574	10098
6	982A	THR	3.02	10577	2210
7	982B	THR	3.77	10567	4844
8	985B	ILE	3.72	10576	4862
9	985B	ILE	3.49	10542	4861
10	985C	ILE	3.39	10558	7495
11	985D	ILE	3.41	10564	10129
12	985D	ILE	3.88	10565	10130

PLIP HBonds interaction // Pi- Stacking // Pi-Pi

Hydrogen Bonds

Index	Residue	AA	Distance H-A	Distance D-A	Donor Angle	Protein donor?	Side chain	Donor Atom	Acceptor Atom
1	983B	VAL	3.21	3.73	115.11	×	×	10555 [Nam]	4848 [O2]

π-Stacking

Index	Residue	AA	Distance	Angle	Offset	Stacking Type	Ligand Atoms
1	981A	TYR	5.09	70.50	1.99	T	10548, 10571, 10572, 10573, 10574, 10575
2	981C	TYR	4.13	25.91	1.46	P	10552, 10566, 10567, 10568, 10569, 10570

Yasara Interactions

Hydrophobic Interaction Analysis
================================

Residue MET A 978 :
to Residue LIG Z 1 : 2 interactions with strength 0.820
Residue TYR A 981 :
to Residue LIG Z 1 : 4 interactions with strength 1.703
Residue TYR B 981 :
to Residue LIG Z 1 : 6 interactions with strength 2.094
Residue THR B 982 :
to Residue LIG Z 1 : 2 interactions with strength 1.105
Residue GLY B 984 :
to Residue LIG Z 1 : 2 interactions with strength 0.685
Residue ILE B 985 :
to Residue LIG Z 1 : 2 interactions with strength 1.115
Residue TYR C 981 :
to Residue LIG Z 1 : 11 interactions with strength 3.900
Residue ILE C 985 :
to Residue LIG Z 1 : 1 interactions with strength 0.705
Residue MET D 978 :
to Residue LIG Z 1 : 1 interactions with strength 0.016
Residue TYR D 981 :

PiPi / CationPi / ionic

PiPi Interaction Analysis
==========================

Residue TYR A 981 :
to Residue LIG Z 1 : 1
interactions with strength 1.000
Residue TYR B 981 :
to Residue LIG Z 1 : 4
interactions with strength 2.219
Residue TYR C 981 :
to Residue LIG Z 1 : 5
interactions with strength 2.146
Residue TYR D 981 :
to Residue LIG Z 1 : 1
interactions with strength 0.098
4 interactions listed.

CationPi Interaction Analysis

to Residue LIG Z 1 : 1 interactions with strength 0.971<br>Residue ILE D 985 :<br>to Residue LIG Z 1 : 2 interactions with strength 0.348<br>Residue LIG Z 1 :<br>in Residue LIG Z 1 : 8 interactions with strength 3.165<br>12 interactions listed.	===========================<br>==<br><br>0 interactions listed.<br><br><br>Ionic Interaction Analysis<br>==========================<br><br>0 interactions listed.
**Hydrogen Bond Analysis**	

**INNER MOUTH-PORE**	
**COMPD. 35**– 79trans_mouthpore_12.pdb	**Contactos a 4 A**
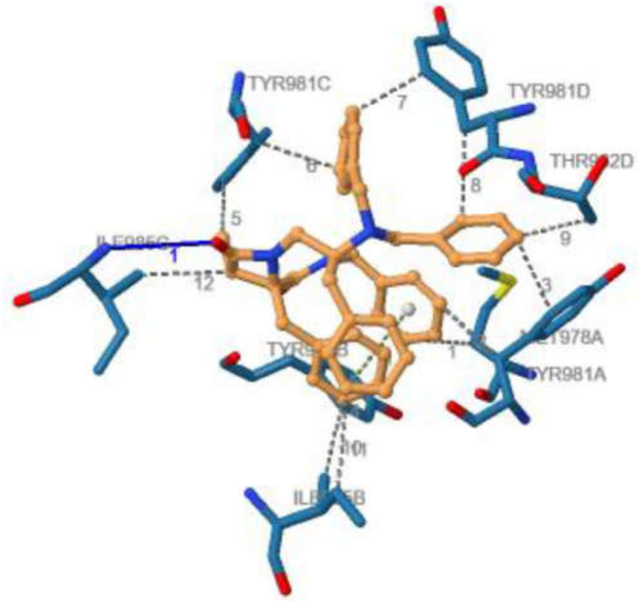	Contact analysis<br>================<br>Residue LIG Z 1 :<br>Contacts to Residue MET A 978 :<br>50<br>Contacts to Residue TYR A 981 : 77<br>Contacts to Residue THR A 982 : 16<br>Contacts to Residue VAL A 983 : 8<br>Contacts to Residue GLY A 984 : 2<br>Contacts to Residue ILE A 985 : 11<br>Contacts to Residue MET B 978 :<br>31<br>Contacts to Residue TYR B 981 : 86<br>Contacts to Residue THR B 982 : 14<br>Contacts to Residue VAL B 983 : 3<br>Contacts to Residue ILE B 985 : 43<br>Contacts to Residue TRP B 994 : 1<br>Contacts to Residue MET C 978 :<br>28<br>Contacts to Residue PHE C 979 : 1<br>Contacts to Residue TYR C 981 : 57<br>Contacts to Residue THR C 982 : 9<br>Contacts to Residue VAL C 983 : 1<br>Contacts to Residue GLY C 984 : 1<br>Contacts to Residue ILE C 985 : 29<br>Contacts to Residue MET D 978 :<br>22<br>Contacts to Residue PHE D 979 : 5<br>Contacts to Residue TYR D 981 : 69<br>Contacts to Residue THR D 982 : 26<br>Contacts to Residue ILE D 985 : 3<br>24 contacts listed.
**PLIP hydrophobic interactions**	

Hydrophobic Interactions ····

Index	Residue	AA	Distance	Ligand Atom	Protein Atom
1	978A	MET	3.96	10578	2173
2	981A	TYR	3.30	10580	2196
3	981A	TYR	3.35	10559	2199
4	981B	TYR	3.41	10573	4833
5	981C	TYR	3.39	10544	7467
6	981C	TYR	3.26	10565	7464
7	981D	TYR	3.13	10563	10101
8	981D	TYR	3.45	10557	10098
9	982D	THR	3.53	10559	10112
10	985B	ILE	3.73	10573	4862
11	985B	ILE	3.56	10568	4861
12	985C	ILE	3.45	10540	7496

PLIP HBonds interaction // Pi- Stacking // Pi-Pi

Hydrogen Bonds

Index	Residue	AA	Distance H-A	Distance D-A	Donor Angle	Protein donor?	Side chain	Donor Atom	Acceptor Atom
1	985C	ILE	3.12	3.93	140.95	✓	×	7490 [Nam]	10582 [O2]

π-Stacking ···· ····

Index	Residue	AA	Distance	Angle	Offset	Stacking Type	Ligand Atoms
1	981B	TYR	4.76	71.07	1.45	T	10556, 10577, 10578, 10579, 10580, 10581

Yasara Interactions	PiPi / CationPi / ionic
Hydrophobic Interaction Analysis<br>================================<br>Residue MET A 978 :<br>to Residue LIG Z 1 : 2 interactions with strength 0.949<br>Residue TYR A 981 :<br>to Residue LIG Z 1 : 2 interactions with strength 2.383<br>Residue MET B 978 :<br>to Residue LIG Z 1 : 2 interactions with strength 0.106<br>Residue TYR B 981<br>to Residue LIG Z 1 : 4 interactions with strength 2.280<br>Residue ILE B 985 :<br>to Residue LIG Z 1 : 4 interactions with strength 1.980<br>Residue TYR C 981 :<br>to Residue LIG Z 1 : 1 interactions with strength 0.205<br>Residue ILE C 985 :<br>to Residue LIG Z 1 : 1 interactions with strength 0.449<br>Residue TYR D 981 :<br>to Residue LIG Z 1 : 2 interactions with strength 0.934<br>Residue THR D 982 :<br>to Residue LIG Z 1 : 1 interactions with strength 0.012<br>Residue LIG Z 1 :	PiPi Interaction Analysis<br>===========================<br>Residue TYR A 981 :<br>to Residue LIG Z 1 : 2 interactions with strength 1.034<br>Residue TYR B 981 :<br>to Residue LIG Z 1 : 4 interactions with strength 3.749<br>Residue TYR D 981 :<br>to Residue LIG Z 1 : 1 interactions with strength 0.060<br>Residue LIG Z 1 :<br>in Residue LIG Z 1 : 2 interactions with strength 2.082<br>4 interactions listed.<br><br>CationPi Interaction Analysis<br>=============================<br><br>0 interactions listed.

<table>
<tr><td>in Residue LIG Z 1 : 10 interactions with strength 7.265
10 interactions listed.</td><td>Ionic Interaction Analysis
==========================

0 interactions listed.</td></tr>
<tr><td>Hydrogen Bond Analysis</td><td></td></tr>
</table>

<table>
<tr><td colspan="2">INNER MOUTH-PORE</td></tr>
<tr><td>COMPD. 37 -- comp55_mouthpore_22.pdb</td><td>Contactos a 4 A</td></tr>
<tr><td>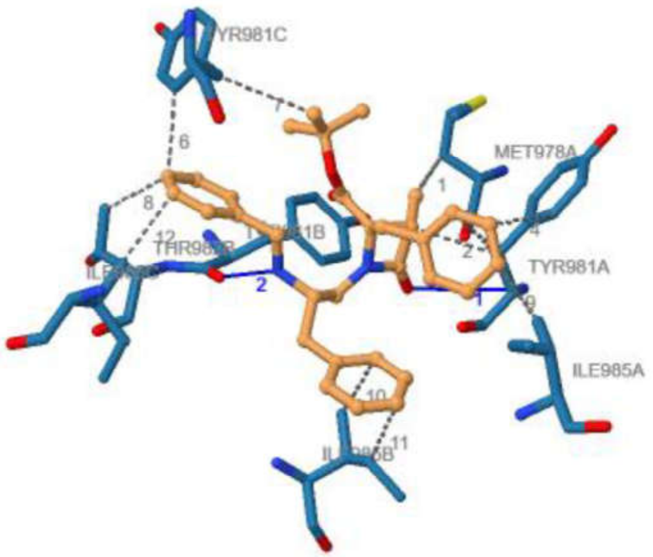
</td><td>Contact analysis
================
Residue LIG Z 1 :
Contacts to Residue MET A 978 : 51
Contacts to Residue PHE A 979 : 1
Contacts to Residue TYR A 981 : 94
Contacts to Residue THR A 982 : 14
Contacts to Residue VAL A 983 : 14
Contacts to Residue GLY A 984 : 14
Contacts to Residue ILE A 985 : 20
Contacts to Residue MET B 978 : 61
Contacts to Residue PHE B 979 : 5
Contacts to Residue TYR B 981 : 96
Contacts to Residue THR B 982 : 36
Contacts to Residue VAL B 983 : 5
Contacts to Residue GLY B 984 : 3
Contacts to Residue ILE B 985 : 74
Contacts to Residue VAL B 986 : 1
Contacts to Residue MET C 978 : 39
Contacts to Residue TYR C 981 : 48
Contacts to Residue ILE C 985 : 22
Contacts to Residue TRP C 994 : 3</td></tr>
</table>

	**Contacts to Residue MET D 978 : 13**<br>**Contacts to Residue TYR D 981 : 1**<br>**Contacts to Residue THR D 982 : 6**<br>**22 contacts listed.**

PLIP hydrophobic interactions

Hydrophobic Interactions

Index	Residue	AA	Distance	Ligand Atom	Protein Atom
1	978A	MET	3.90	10542	2173
2	981A	TYR	3.13	10537	2196
3	981A	TYR	3.69	10566	2196
4	981A	TYR	3.18	10567	2199
5	981B	TYR	3.43	10556	4830
6	981C	TYR	3.02	10558	7467
7	981C	TYR	3.70	10571	7464
8	982B	THR	3.10	10558	4844
9	985A	ILE	3.58	10568	2228
10	985B	ILE	3.35	10561	4862
11	985B	ILE	3.28	10563	4861
12	985C	ILE	3.03	10559	7496

PLIP HBonds interaction // Pi- Stacking // Pi-Pi

Index	Residue	AA	Distance H-A	Distance D-A	Donor Angle	Protein donor?	Side chain	Donor Atom	Acceptor Atom
1	981A	TYR	3.65	4.07	108.40	✔	×	2192 [Nam]	10543 [O2]
2	981B	TYR	2.09	2.58	107.35	×	×	10547 [N3]	4829 [O2]

**Yasara Interactions**	**PiPi / CationPi / ionic**
Hydrophobic Interaction Analysis<br>================================<br><br>Residue MET A 978 :<br>to Residue LIG Z 1 : 2 interactions with strength 1.225<br>Residue TYR A 981 :<br>to Residue LIG Z 1 : 7 interactions with strength 2.726<br>Residue GLY A 984 :<br>to Residue LIG Z 1 : 1 interactions with strength 0.217<br>Residue ILE A 985 :<br>to Residue LIG Z 1 : 1 interactions with strength 0.115<br>Residue TYR B 981 :<br>to Residue LIG Z 1 : 1 interactions with strength 0.517<br>Residue ILE B 985 :<br>to Residue LIG Z 1 : 3 interactions with strength 0.756<br>Residue MET C 978 :<br>to Residue LIG Z 1 : 2 interactions with strength 0.696<br>Residue TYR C 981 :<br>to Residue LIG Z 1 : 2 interactions with strength 0.413<br>Residue MET D 978 :<br>to Residue LIG Z 1 : 1 interactions with strength 0.555	PiPi Interaction Analysis<br>=========================<br><br>Residue TYR C 981 :<br>to Residue LIG Z 1 : 1 interactions with strength 1.000<br>Residue LIG Z 1 :<br>in Residue LIG Z 1 : 1 interactions with strength 1.000<br>2 interactions listed.<br><br>CationPi Interaction Analysis<br>============================<br><br>0 interactions listed.<br><br>Ionic Interaction Analysis

Residue LIG Z 1 :<br>in Residue LIG Z 1 : 4 interactions with strength 1.145<br>10 interactions listed.	==========================<br><br>0 interactions listed.
**Hydrogen Bond Analysis**	

References

1. Gonzalez Muniz, R.; Perez Ma. Jesus, de V.; Bonache de Marcos, M.A.; Ferrer Montiel, A.; Fernandez Carvajal, A.; De la Torr, e. R. Heterocyclic compounds as TRPM8 channel antagonists and uses thereof. *WO 2017005950* 2017, WO 2017005950.

2. Martin-Escura, C.; Medina-Peris, A.; Spear, L.A.; de la Torre Martinez, R.; Olivos-Ore, L.A.; Barahona, M.V.; Gonzalez-Rodriguez, S.; Fernandez-Ballester, G.; Fernandez-Carvajal, A.; Artalejo, A.R.; et al. β-Lactam TRPM8 Antagonist RGM8-51 Displays Antinociceptive Activity in Different Animal Models. *Int. J. Mol. Sci.* **2022**, *23*, 2692, doi:10.3390/ijms23052692.
